# Mapping molecular subtype specific alterations in breast cancer brain metastases identifies clinically relevant vulnerabilities

**DOI:** 10.1038/s41467-022-27987-5

**Published:** 2022-01-26

**Authors:** Nicola Cosgrove, Damir Varešlija, Stephen Keelan, Ashuvinee Elangovan, Jennifer M. Atkinson, Sinéad Cocchiglia, Fiona T. Bane, Vikrant Singh, Simon Furney, Chunling Hu, Jodi M. Carter, Steven N. Hart, Siddhartha Yadav, Matthew P. Goetz, Arnold D. K. Hill, Steffi Oesterreich, Adrian V. Lee, Fergus J. Couch, Leonie S. Young

**Affiliations:** 1 Endocrine Oncology Research Group, Department of Surgery, RCSI University of Medicine and Health Sciences, Dublin, Ireland; 2 WCRC, UPMC Hillman Cancer Center, Magee-Womens Research Institute, University of Pittsburgh, Pittsburgh, PA USA; 3 Genomic Oncology Research Group, Royal College of Surgeons in Ireland, Dublin, Ireland; 4 Department of Laboratory Medicine and Pathology, Mayo Clinic, Rochester, MN USA; 5 Department of Quantitative Sciences Research, Mayo Clinic, Rochester, MN USA; 6 Department of Oncology, Mayo Clinic, Rochester, MN USA; 7 Department of Pharmacology and Chemical Biology, University of Pittsburgh, Pittsburgh, PA USA

**Keywords:** Metastasis, Gene regulatory networks, Breast cancer

## Abstract

The molecular events and transcriptional plasticity driving brain metastasis in clinically relevant breast tumor subtypes has not been determined. Here we comprehensively dissect genomic, transcriptomic and clinical data in patient-matched longitudinal tumor samples, and unravel distinct transcriptional programs enriched in brain metastasis. We report on subtype specific hub genes and functional processes, central to disease-affected networks in brain metastasis. Importantly, in luminal brain metastases we identify homologous recombination deficiency operative in transcriptomic and genomic data with recurrent breast mutational signatures A, F and K, associated with mismatch repair defects, *TP53* mutations and homologous recombination deficiency (HRD) respectively. Utilizing PARP inhibition in patient-derived brain metastatic tumor explants we functionally validate HRD as a key vulnerability. Here, we demonstrate a functionally relevant HRD evident at genomic and transcriptomic levels pointing to genomic instability in breast cancer brain metastasis which is of potential translational significance.

## Abstract (web summary)

The molecular landscape of breast cancer brain metastases (BCBM) is still understudied, especially for different breast cancer subtypes. Here, the authors characterise subtype-specific BCBMs using genomics and transcriptomics and identify homologous recombination deficiency as a key therapeutic vulnerability.

## Introduction

Breast cancer brain metastases (BCBM) are a frequent and aggressive form of metastatic spread, with treatment options limited for each of the clinically relevant breast cancer subtypes^1^. Breast cancer cells exhibit exceptional plasticity, capable of adapting to sequential bouts of therapeutic pressure, as well as the vastly changing microenvironmental landscape. These adaptations can be immediate or delayed, often depending on whether tumors are ER-positive or ER-negative^2^. Breast cancer brain metastases diverge from their primary breast tumors both genomically and phenotypically. At a most basic level, this is observed in frequent clinical and molecular subtype switching reported in brain metastases^3,4^. The receptor discordance is most prominent in luminal (ER-positive) tumors that may inform subtype-directed therapeutic approaches. Furthermore, despite differences in the rate of BCBM recurrence amongst different breast cancer subtypes, the presentation of BCBM carries with it the highest risk of death which remains comparable between ER-positive and ER-negative tumors^2^. The molecular diversity of BCBM and its relationship to tumor subtype has not been elucidated, especially in the context of BCBM originating from luminal tumors. While luminal tumors are less aggressive, they are by far the largest molecular subtype and therefore represent a significant number of metastatic cases and deaths^1,5^, underscoring the necessity for a greater understanding of molecular drivers and the underlying biology.

Numerous studies have made use of gene expression profiling of triple-negative and HER2+ve BCBM-homing cell line models to identify drivers of various BCBM-related processes, some of which are associated with brain relapse-free survival in primary tumors^6–11^. On the other hand, investigations exploring the genomic landscape of resected BCBM tumors have attempted to investigate putative driver mutations, clonality, and genetic divergence. Acquired driver genomic alterations in BCBM predominantly consist of the HER, PI3K, and cyclin-dependent kinase (CDK) pathways; many of which are enriched compared to the primary tumor^12–14^. Although this general strategy has classified potentially clinically informative adaptations, only a handful of studies have investigated these mutations in experimental models or in patients, especially in the context of all breast tumor subtypes. As such, there remains an uncertainty about the functional relevance of these events and their specificity for BCBM.

In this work, as part of a multi-institutional effort, we have profiled genetic and transcriptomic features of longitudinal patient-matched BCBMs with corresponding comprehensive clinical annotation including full treatment history and patient outcomes at each step of progression. Whilst the genomic and transcriptomic landscape of BCBM is widespread it converges on several key pathways and effectors demonstrating the value of interrogating these processes collectively. In this study, our cohort allowed us to characterize and map breast cancer subtype-specific BCBM alterations through interrogation of DNA and RNA-sequencing data combined with a network analysis-based approach. DNA repair pathway defects, including homologous recombination deficiency (HRD), are extensively profiled and functionally validated in luminal BCBMs.

## Results

### Subtype-specific BCBM transcriptome

**Fig. 1:**
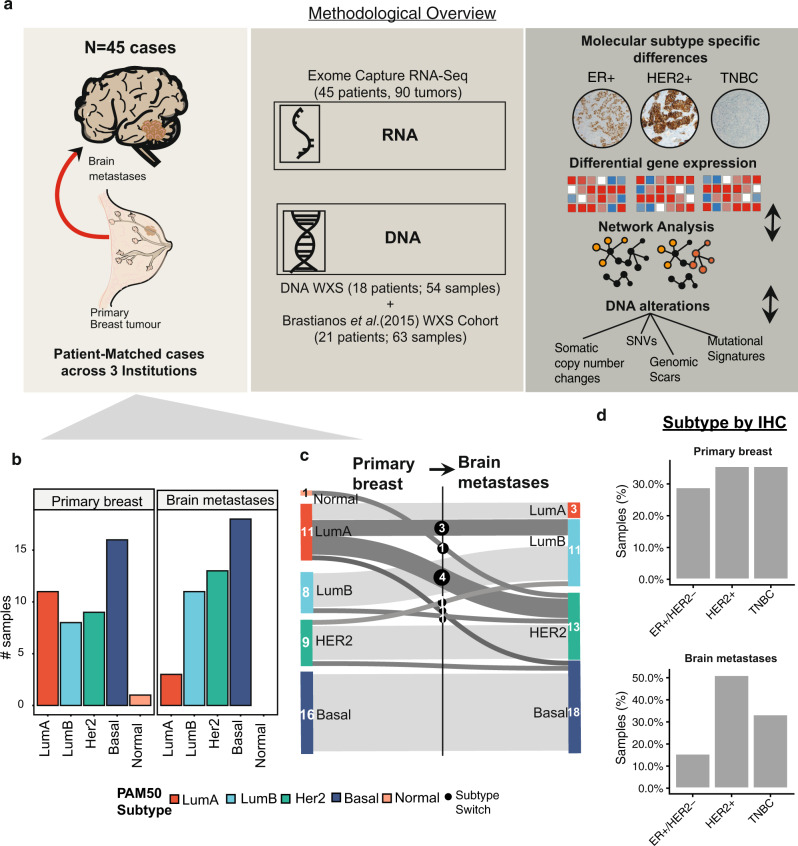
Methodological overview and BCBM subtyping. **a**, **b** Graphical methodological overview of the subtype-specific analysis of exome capture RNA-Seq (45 cases; 90 samples) and DNA whole-exome sequencing for 18/45 cases. **c** Bar chart shows the frequency of intrinsic molecular subtype: Luminal A, B, Her2-enriched, Basal-like, and Normal for primary breast and brain metastatic tumors (left). The intrinsic molecular subtype of each tumor was called by applying the PAM50 subtype predictor to gene expression data, adjusted for sequencing center batch-driven effect. Sankey flow diagram of molecular sample switching from primary breast tumors to brain metastases (Right). Dark gray edges of the flow diagram represent those samples that switched intrinsic molecular subtype. **d** Bar chart of percent samples annotated as either ER^+^/HER2^+^, HER2^+^, TNBC tumor subtype, defined based on immunohistochemistry (IHC) status for ER, PR, and HER2 genes when available for 90 samples. Source data are provided as a Source Data file.

**Fig. 2:**
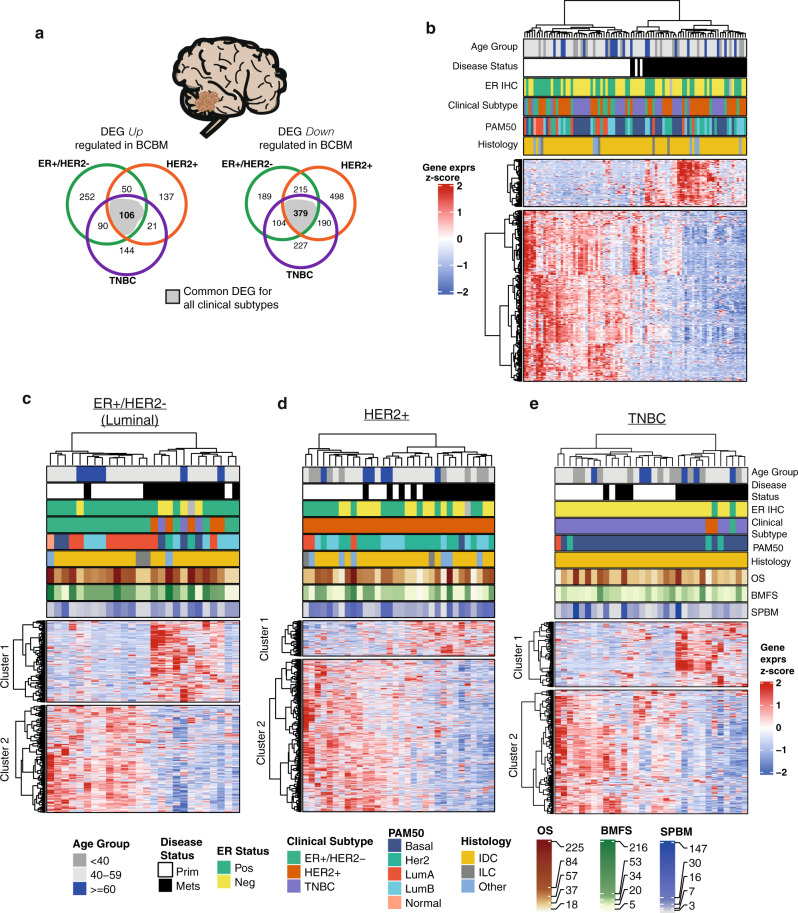
Clinical subtype-specific differential gene expression in BCBM. **a** Venn diagram representations of significantly (log2FC ± 2.0; FDR < 0.05) up (top) and downregulated (bottom) genes in BCBM from a clinical subtype-specific differential gene expression analysis. Differentially expressed genes (DEG) common to all subtype-specific analysis highlighted in gray (up: *n* = 106 genes, down: *n* = 379 genes). Clinical molecular subtype of the primary breast tumor: ER^+^/HER2^−^ (green), HER2^+^ (orange), TNBC (purple). **b** Heatmap of hierarchical clustering of DEG common to all subtypes dysregulated in BCBM. Gene expression *z*-scores: upregulated in BCBM (red), downregulated in BCBM (blue). Unsupervised sample clustering (dendrogram) shows a clear separation between primary breast tumors (white) and BCBM tumors (black), with overall global loss of gene expression in BCBM. **c**–**e** Heatmaps of unsupervised hierarchical clustering of clinical subtype-specific BCBM DEGs. From left to right: Luminal, HER2^+^ and TNBC subtype respectively. Gene expression *z*-scores: upregulated in BCBM (red), downregulated in BCBM (blue). Common legend for all heatmaps (**b**–**e**). OS overall survival; BMFS brain metastases-free survival; SPBM survival post brain metastasis all in months. Disease status indicates sample type (primary = primary tumor or Mets = BCBM). IDC invasive ductal carcinoma; ILC invasive lobular carcinoma.

To date, BCBM molecular drivers have not been characterized for each individual clinical breast subtype potentially missing key insights into the biology and heterogeneity of the disease. To map subtype-specific alterations in BCBM, we analyzed patient-matched primary breast and brain metastatic RNA and DNA samples from a cohort of 45 and 39 patients respectively (Fig. 1a). 13 ER^+^/HER2^−^ (which we designate as luminal) (29%), 16 HER2^+^ (ER^+/−^) (35.5%) and 16 TNBC (35.5%) tumors underwent RNA-sequencing and are presented with fully annotated clinicopathological characteristics (Table 1, Supplementary Figs. 1–3, Supplementary Data 1). Consistent with previous reports^3,4,15^, we observed both intrinsic molecular subtype switching and clinical subtype switching from primary breast to BCBM for ~27% (12/45) and 22% (10/45) cases respectively (Fig. 1b–d and Supplementary Data 2, Supplementary Fig. 4). We analyzed the tumor pairs with regard to clinical subtypes which exhibited discrete transcriptional programs (differentially expressed in BCBM compared to patient-matched primaries, log2FC ± 2.0; adjusted *P*-value < 0.05) (Fig. 2a and Supplementary Data 3–5). We identified commonly differentially expressed genes (106 up-; 379 downregulated in BCBM, Supplementary Data 6) enriched for pathways associated with the brain tumor microenvironment (GSEA; FDR < 0.25; NES ± 1.0), including *GFAP*, glial fibrillary acidic protein (a marker of reactive astrocytes), gene targets of *NR2E1* (TLX), nuclear receptor subfamily 2 group E member 1(encoded protein regulates adult neural stem cell proliferation), and *PTPRC*, protein tyrosine phosphatase receptor C (signaling molecules that regulate multiple cellular processes^16–18^) (Fig. 2b and Supplementary Data 7).

**Table 1:** Demographic and clinical characteristics of brain metastases cohort.

Clinical subtype	ER^+^/HER2^−^	HER2^+^	TNBC
Number of patients	13	16	16
Age at diagnosis*	53 [40, 63]	50 [26, 67]	46 [26, 66]
Age group (%)			
<40	0 (0.0)	4 (25.0)	5 (31.2)
40–59	10 (76.9)	9 (56.2)	8 (50.0)
≥60	3 (23.1)	3 (18.8)	3 (18.8)
Vital status (%)			
Alive	1 (7.7)	1 (6.2)	2 (12.5)
Dead	12 (92.3)	15 (93.8)	14 (87.5)
Overall survival*	74 [32, 223]	58 [18, 225]	42 [18, 173]
BMFS*	53 [12, 216]	31 [5, 151]	25 [13, 89]
SPBM*	18 [5, 65]	22 [3, 74]	12 [3, 147]
Other metastases (%)			
Yes	10 (76.9)	10 (62.5)	8 (50.0)
No	3 (23.1)	6 (37.5)	8 (50.0)
Histological subtype (%)			
IDC	11 (84.6)	12 (75.0)	16 (100.0)
ILC	1 (7.7)	1 (6.2)	0 (0.0)
Other	1 (7.7)	3 (18.8)	0 (0.0)
PAM50 subtype (%)			
LumA	7 (53.8)	3 (18.8)	1 (6.2)
LumB	3 (23.1)	5 (31.2)	0 (0.0)
Her2	0 (0.0)	8 (50.0)	1 (6.2)
Basal	2 (15.4)	0 (0.0)	14 (87.5)
Normal	1 (7.7)	0 (0.0)	0 (0.0)

*BMFS* brain metastases-free survival in months (time from primary breast diagnosis to brain metastatic disease diagnosis), *SPBM* survival post brain metastases in months (time from brain metastases diagnosis to death or follow up), *IDC* invasive ductal carcinoma, *ILC* invasive lobular carcinoma,

*Median [min, max].

In clinical subtype-specific transcriptome analysis, unsupervised clustering identified distinct BCBM expressed gene clusters (Fig. 2c–e, see “Methods” section). GSEA revealed luminal subtype (ER^+^/HER2^−^)-specific gene expression changes in BCBM, enriched for downregulated NOTCH, AKT, and p53 signaling pathways, with upregulation of myogenesis (KLF2) and response to oxygen associated pathways (Supplementary Data 8). HER2^+^ BCBM show downregulation of focal adhesion cellular processes, ECM, and members of the neuroactive ligand-receptor signaling pathway. We found a significant positive enrichment for metabolic and hypoxia associated function in HER2^+^ BCBM, driven primarily by the upregulation of *ALDOA*, *GPI,* and *ENO1* genes. TNBC BCBM demonstrate downregulation of ITGAL, cytotoxic T cell, and interferon-gamma-associated pathways, with upregulation of cell cycle and LEF1 transcription factor WNT signaling (Supplementary Data 8).

**Fig. 3:**
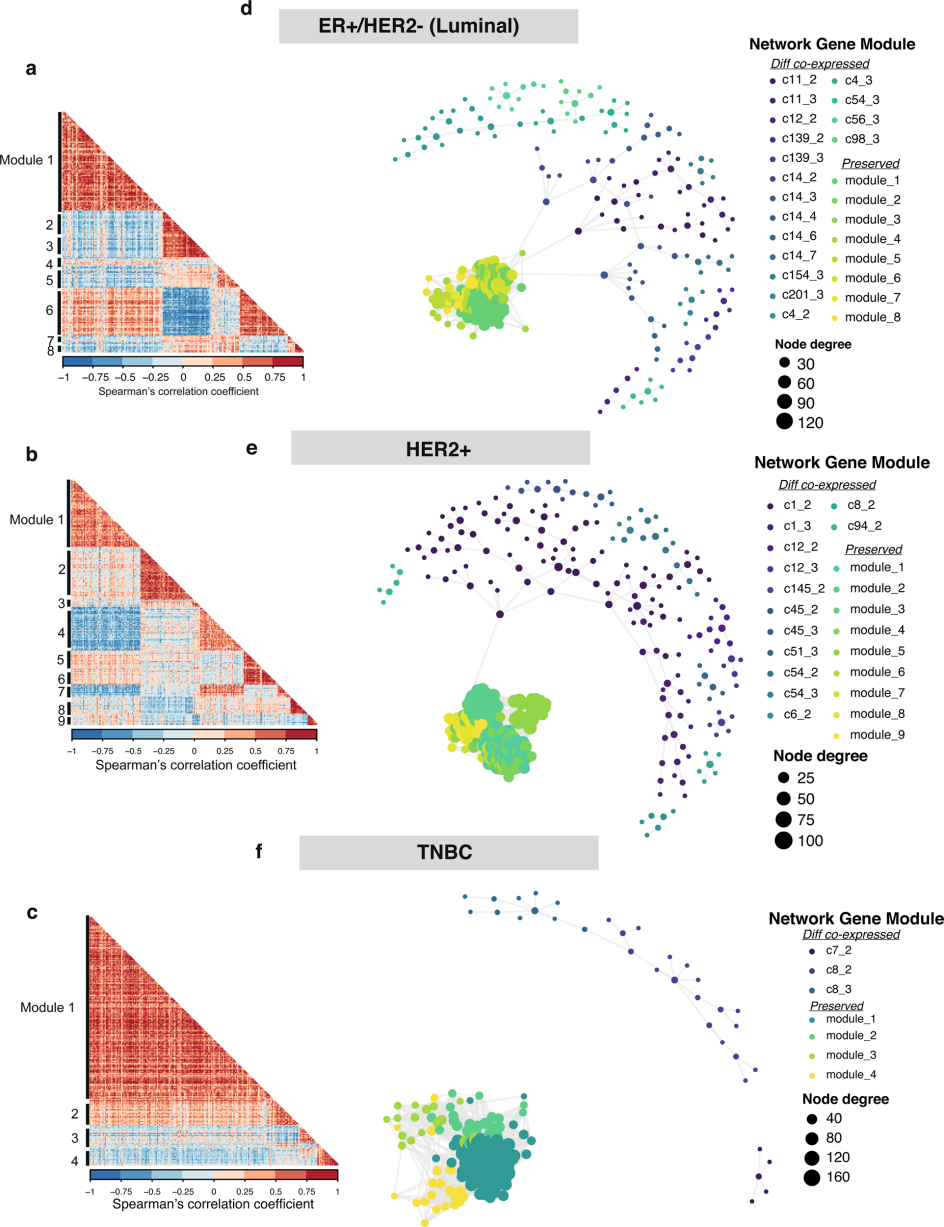
Clinical subtype-specific gene networks in BCBM. **a**–**c** Correlation heatmaps of gene-gene correlation coefficients, i.e., the strength and direction of gene co-expression in each clinical subtype, from left to right, Luminal, HER2^+^, and TNBC. Module membership is labeled. Positive spearman correlation coefficient colored in red, with negative correlation coefficient colored in blue. (Benjamini–Hochberg (BH) adjusted *P*-value < 0.05). **d**–**f** Network plots of clinical subtype-specific gene co-expression and differential co-expression networks identified for Luminal, HER2^+^, and TNBC subtype (Left-right). For each network (**d**–**f**) gene nodes are colored according to gene module membership, with gene node size proportional to node degree value. Larger degree values indicate those genes which are highly connected to other genes in the network and are most likely hub genes.

**Fig. 4:**
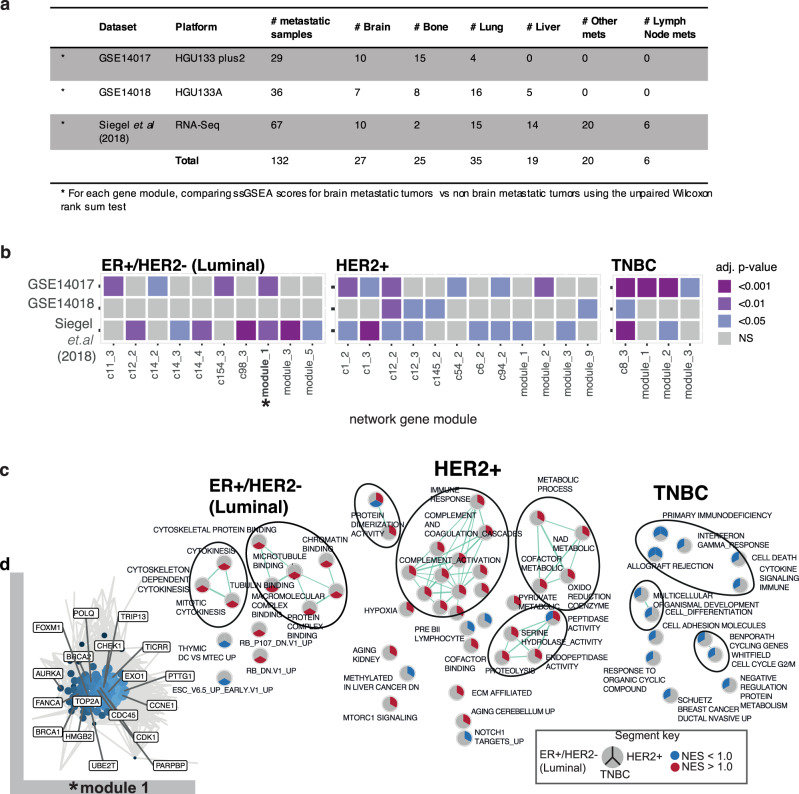
Brain-specific gene networks. **a** Summary of the breast cancer brain metastases publicly available data sets utilized for ssGSEA testing of gene co-expression network modules. **b** Tile plot of those significantly enriched network gene modules for each subtype in independent breast cancer metastases gene expression data sets. Tile color corresponds to adjusted *P*-value from testing for differences in ssGSEA gene module scores between brain metastatic samples versus other metastatic sites (two-sided Wilcoxon rank-sum (Mann–Whitney *U*) test used; Benjamini–Hochberg adjusted *P*-value < 0.05, exact *P* and *Q* values provided in Supplemental Data 10). **c** Enrichment Map plot of functional annotation of significantly enriched gene modules using GSEA and the Molecular Signatures database (MSigDB) (Normalized enrichment score (NES) ±1.0; BH adjusted *P*-value < 0.10). *P*-values are based on a gene-permutation test and adjusted using the Benjamini–Hochberg procedure (see “Methods”, “Gene set enrichment analysis”). Functional pathway term similarity colored by green edges; nodes colored per NES score (red indicates positive enrichment in brain metastases vs primary breast tumors, with blue indicating negative enrichment). **d** Luminal gene network module 1 vignette shown, with manually curated DNA repair-associated genes labeled.

Functionally, genes do not act in isolation and as such, we next prioritized identification of BCBM gene co-expression networks for each clinical subtype using the WGCNA framework (see “Methods” section, Supplementary Fig. 4). We identified 8 gene co-expression modules (*n* = 197 genes) in luminal (ER^+^/HER2^−^), 9 modules (*n* = 231 genes) in HER2^+^ and 4 modules (*n* = 229 genes) TNBC subtypes, all of which were present in both primary tumor and BCBM (Fig. 3a–c and Supplementary Fig. 5). Focusing on functionally related gene networks altered with BCBM, differential gene co-expression network analysis (DGCA), further defined 17 luminal (*n* = 164 genes), 13 HER2^+^ (*n* = 186 genes) and 3 TNBC (*n* = 34 genes) differential gene co-expression modules (Supplementary Figs. 5 and 6). We observe overall, TNBC gene networks are less divergent compared to luminal and HER2^+^ subtypes, with network connectivity strongly driven by gene networks present both in primary and BCBM (Fig. 3d–f). This could partly be due to the heterogeneity present within the TNBC tumors themselves as evidenced by their Lehman subtyping classifications (Supplementary Data 2). Most of the network structure captured here reflects the often-observed tumor heterogeneity within clinical subtypes. To query whether these modules were BCBM-specific rather than a general metastatic alteration we analyzed several breast gene expression data sets with multiple annotated metastatic sites including brain, bone, lung, liver, and other sites^14,19,20^. By comparing ssGSEA score for each gene module in the brain versus all other metastatic sites notably, we found that ~79% (26/33; adjusted *P*-value < 0.05) of the gene modules were significantly enriched in BCBM over other sites (Fig. 4a, b and Supplementary Fig. 7, Supplementary Data 9).

Pathway activity of these modules recapitulated some known characteristics of each clinical subtype, but we also observed alterations in pathways previously not reported (Fig. 4c and Supplementary Data 10). HER2^+^ subtype brain-specific gene networks show downregulation of TNF-α/NFK-α, INHBA-mediated immune response, ECM proteins, and mammary stem cell-related pathways. Consistent and complementary to differential gene expression in HER2^+^ BCBM versus matched primary breast tumor, one module (module_1) was brain-specific and enriched for ENO1-mediated metabolic reprogramming and mTORc related signaling. The second-largest HER2^+^ BCBM-specific gene module (module_2) shows upregulation of complement cascade (C1QA/B/C), with depletion of NOTCH1 (BIRC3, CD3G, CD74, CD2), MYC targets (PTPRC, CD2, CD74), and T cell receptor signaling. For patients with TNBC, the BCBM-specific gene module (c8_3) is strongly associated with SERPINF1, INHBA-enriched cell death, and differentiation function. TNBC gene module 1 was enriched for pathways related to interferon-gamma response, cell cycle G2/M phase (*VCAM1*, *IDO1*), and T cell differentiation (*CARD11*, *LCK, B2M*) function (Fig. 4c). Notably, in the luminal cohort, a BCBM-specific gene co-expression network module (module 1) genes are enriched for mitotic cytokinesis, *p53* signaling, *RB1* gene, and *AURKA* related cell proliferation function and *BRCA1*-mediated cell cycle regulation (gene ontology tubulin/chromatin binding) (Fig. 4c). Indeed, annotating co-expression module genes according to the Drug-Gene Interaction database (DGIdb)^21^ categories revealed the highest proportion of DNA repair genes belonged to the luminal subtype network genes (Supplementary Fig. 8). Moreover, further manual annotation of luminal module 1 network genes with DGIdb categories revealed several known DNA repair pathway genes including *BRCA1*, *BRCA2*, *CHEK1,* and *AURKA* (Fig. 4d). Though germline and somatic mutations in *BRCA1* and *BRCA2* genes are known to be associated with HRD, here, transcriptomic network irregularities in BRCA driven pathways could also be utilized to identify tumors potentially harboring irregularities in these pathways. Taken together, harmonization of subtype-specific approaches exposes transcriptome network irregularities revealing hard-to-detect and potentially biologically significant networks.

### Homologous recombination deficiency is enriched in brain metastases

**Fig. 5:**
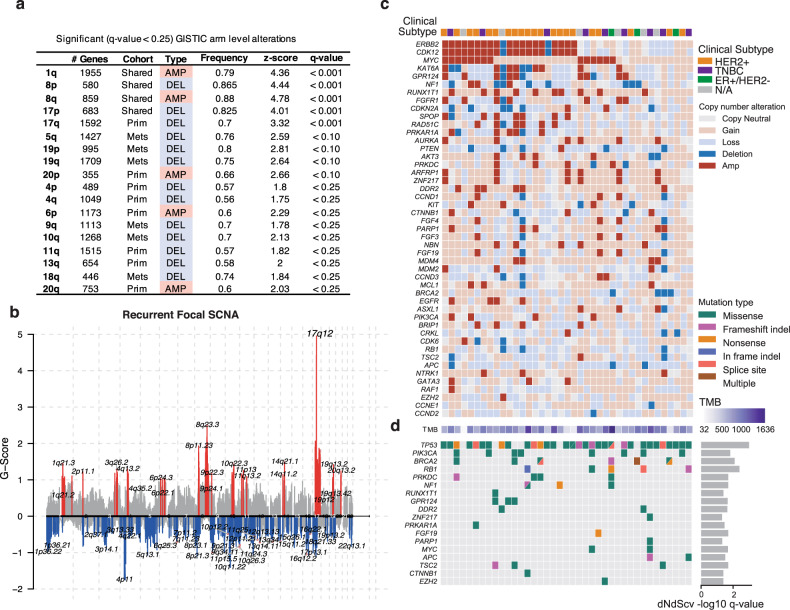
Genomic landscape of breast to brain metastasis. **a** Summary of GISTIC broad arm level alterations shared between primary and BCBM, exclusive to primary (Prim) or exclusive to BCBM (Mets) annotated by chromosome arm. Significant if *q*-value < 0.25. *P*-values are based on a gene-permutation test and adjusted using the Benjamini–Hochberg procedure. Exact *P* and *Q* values are provided in Supplemental Data 12. (see “Methods”, “Identification of recurrent somatic copy number alterations”). **b** GISTIC2 chromPlot shows the frequency and magnitude (*G*-Score) of recurrent somatic copy number alterations (SCNA) identified in BCBM across chromosomes 1–22 (left-right). Copy number (CN) gains and amplifications colored in red, with CN loss and deletions colored in blue. Significant (FDR < 0.10) GISTIC2 recurrent SCNA labeled. **c**, **d** Oncoplot of recurrent SCNA and SNVs detected in brain metastatic tumors. Samples annotated by brain metastatic clinical subtype and tumor mutational burden (TMB). SNVs annotated by their associated dNdScv –log10 *q*-value, from testing for SNV’s positively selected for in brain metastases and likely a driver mutation.

We next sought to determine whether DNA alterations in BCBM impacted comparable pathways. We performed WXS on 18/45 of BCBM cases (18 trios consisting of BCBM and matched primary tumor and normal tissue) and analyzed an additional independent BCBM WXS cohort (*N* = 21 cases)^12^ (Supplementary Data 11). Somatic copy number alteration (SCNA) analysis between patient-matched cases revealed both shared and distinct large-scale amplifications and deletions (*q*-value < 0.001) (Fig. 5a, b and Supplementary Data 12). Notably, arm level amplifications (chr20p, 20q, chr6p; *q*-value < 0.25) were enriched in primary breast tumors, with brain metastasis-specific recurrent arm level alterations enriched for copy number loss and deletions (chr5q, 19p,19q,9q,10q,18q; *q*-value < 0.25) (Supplementary Data 12). Fifteen regions of recurrent focal amplifications (including chr17q12, 8p11.23, 8q23.3, and 20q13.2) versus 47 regions of focal deletions (including chr4p11) were identified as significantly altered in brain metastases (*q*-value < 0.10) (Fig. 5b and Supplementary Data 13, 14). Gene level variant calling identified, copy number changes in BCBM including amplifications in *ERBB2*, *MYC*, *AURKA* with deletions in tumor suppressor genes such as *NF1*, *PTEN*, along with SNVs in *TP53*, *PIK3CA,* and *BRCA2* (Fig. 5c, d and Supplementary Data 15). In most BCBM cases, regions of significant SCNA (both broad and focal alterations) were largely comprised of deletions, potentially indicative of genomic instability. The observed genomic instability in BCBM tumors and in particular the prevalence of deletions, is consistent with probable defects in DNA repair pathway function and maybe reflective of the accumulated treatment history as has been reported elsewhere^22^. In our data set, however, we see no association in terms of types of therapies or number of therapies having an influence on the specific mutational landscape.

**Fig. 6:**
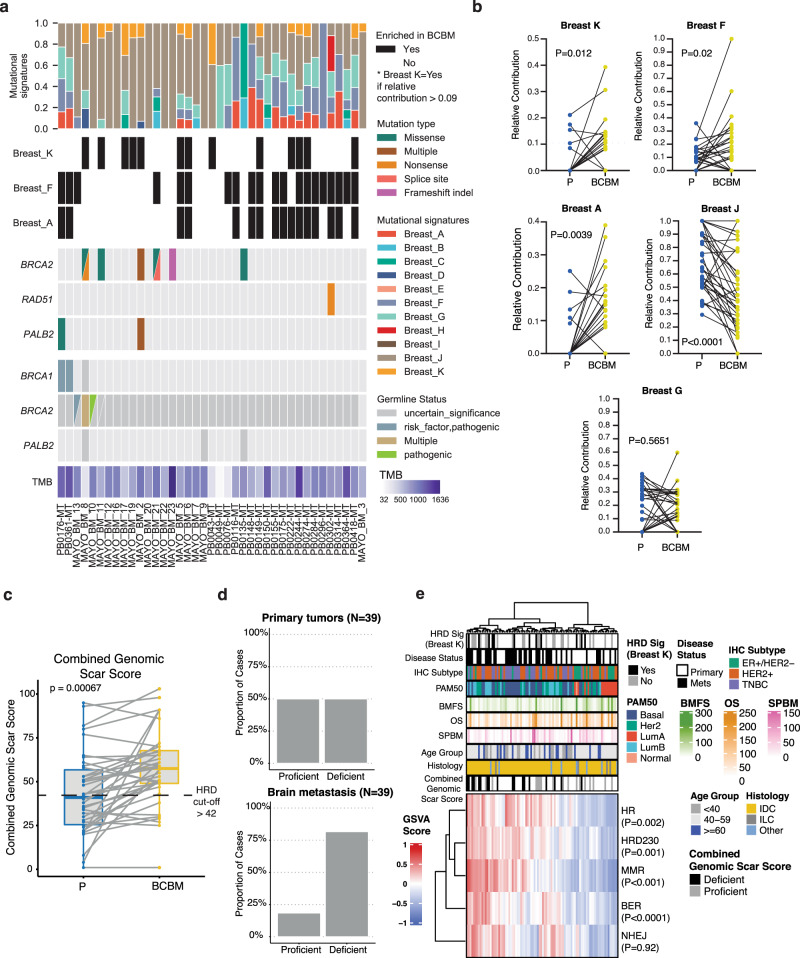
Homologous recombination deficiency is enriched in breast cancer brain metastases. **a** Stacked bar chart of the relative contribution [0–1] of breast cancer mutational signatures (Breast A-K) detected in BCBM (*N* = 39 patients). Black tile plot indicates enrichment in BCBM over patient-matched primary. Tumor mutational burden (TMB), somatic and germline SNV’s detected in BCBM tumors for *BRCA2*, *RAD51,* and *PALB2* genes are displayed. **b** Relative contribution values for mutational signatures significantly altered in BCBM (yellow) compared to matched primary tumor (P) (blue) (paired two-sided Wilcoxon test *P* < 0.05). Mismatch repair (MMR1; Breast A), Ageing associated signature (Breast J), a signature of unknown etiology (Breast F), and HRD-associated signature (Breast K). **c** Boxplot of the combined genomic scar score calculated using allele-specific copy number calling with FACETS from BCBM WXS DNA seq Cohort (*N* = 39 patients). Paired one-sided Wilcoxon test was used to compare HRD score in matched primary breast (blue) and BCBM tumors (yellow). Dashed line indicates HRD cut-off score of 42. The upper and lower limits of the box correspond to the 1st and 3rd quartile score distribution with whiskers extending to 1.5 times the range from top/bottom of the box. **d** HRD status of the primary tumor (top) and brain metastasis (bottom). The charts represent the percentage of tumors that are scored as proficient or deficient in both primary and how this status is altered in BCBM. **e** Heatmap of hierarchical clustering of sample and GSVA pathway scores in RNA-Seq BCBM Cohort (*N* = 90 samples). From top-bottom on the heatmap, GSVA scores for HR, HRD230, MMR KEGG, BER and NHEJ DNA repair pathways from KEGG. (Paired two-sided Wilcoxon test. HR, *P* = 0.0002; HRD230, *P* = 0.0013; MMR, *P* = 0.0001; BER, *P* = 0.0009; NHEJ, *P* = 0.93). OS overall survival; BMFS brain metastases-free survival; SPBM survival post brain metastasis all in months. The ranges are included in the legend. Disease status indicates sample type (primary = primary tumor or Mets = BCBM). Source data are provided as a Source Data file.

We subsequently investigated mutational processes active in BCBM using a recently described organ-specific framework for mutational signature analyses^23^. Overall BCBM tumors were composed of Breast A (RefSigMMR1; mismatch repair deficiency (MMR)), Breast F (RefSig18; reported associated driver mutations *TP53*, *APC*, *NOTCH* and *NFE2L2*), Breast G (RefSig30; TP53 driver mutation associated), Breast K (RefSig3; HRD-related; reported associated driver mutations *BRCA2, TP53, BRCA1, MYC, ARID1, NF1*) and Breast J (RefSig 1; ageing associated; associated driver mutations *TP53, KRAS, CDKN2B, CDKN2A, EGFR, SMA4, APC, BRD4*), with a minority of tumors with Breast D (RefSig MMR2; associated driver mutations *CTNNB1, ALB*), Breast B (RefSig2, *APOBEC*) and Breast C (RefSig13, *APOBEC*; associated driver mutations *TP53, PIK3CA, FAT1*) (Fig. 6a and Supplementary Fig. 9, Supplementary Data 16, 17).

Mutational signatures Breast A (MMR1), Breast K (HRD), and Breast F were significantly enriched in BCBM compared to matched primary breast tumor with the relative contribution of Breast J (Ageing associated) decreased in BCBM (Fig. 6b; paired Wilcoxon rank-sum test; *P* < 0.05). We employed a benchmarking strategy to establish a threshold to define Breast K signature status^23^. Using the defined cut-off of relative contribution greater >0.9, we detected Breast K in 13 out of 39 BCBM of which 9 cases did not have Breast K present in the matched primary tumor indicating a HRD-associated signature gained in BCBM (Fig. 6a and Supplementary Fig. 9c, Supplementary Data 17). Intriguingly, HRD mutational signature Breast K was found in 54% (6/11) of luminal type BCBM independently of somatic or germline BRCA1/2/PALPB2 mutations, with 31% (6/19) in HER2^+^ and 11% (1/9) in triple-negative subtype (Supplementary Data 17). We found that ~21% (6/39) of BCBM cases had the presence of Breast K signature mutually exclusive to the other BCBM enriched mutational signatures, independently of somatic or germline *BRCA1/2/PALPB2* mutations and tumor mutational burden (Fig. 6a and Supplementary Fig. 10; Supplementary Data 17). Of note, we observed one pathogenic germline *BRCA2* mutation and two somatic *BRCA2* and/or *PALB2* mutations in only 2/13 HRD BCBM cases, along with several germline variants of uncertain significance in *BRCA1/2* and *PALB2* genes across all 39 cases which did not associate with the HRD-related signatures we detected (annotated by ClinVar database; Fig. 6a and Supplementary Fig. 10, Supplementary Data 18). Likewise, the transcript levels of *BRCA1*, *RAD51,* and *RAD51C* were largely unaltered in BCBM samples harboring high levels of HRD-related signatures (Supplementary Fig. 9e). Therefore, we conclude that the HRD mutational signature detected here is independent of known germline and somatic *BRCA1/2* and *PALB2* mutations.

To further define the increased presence of HRD in BCBM tumors, we also calculated a combined genomic scar score, a marker of genomic instability associated with a double-strand break (DSB) repair and HRD^24,25^, including HRD loss of heterozygosity (HRD-LOH), large state transitions (LST) and the number of telomeric allelic imbalance (ntAI) (see “Methods” section). The combined “*genomic scar*” score was significantly increased in BCBM compared to matched primary breast tumor (Fig. 6c, d; one-sided paired Wilcoxon rank-sum test, *P* < 0.05). Interestingly, in the BCBM cases where we detect Breast K, 11/13 BCBM samples are also called HR deficient by the genomic scar method (score > 41) (Supplementary Data 17). Collectively, these data are consistent with a model where DNA repair pathways represent a key genomic dependency enriched in luminal and other BCBM and these alterations might endow a survival advantage for breast tumors.

To ascertain whether HRD is functionally represented in the BCBM transcriptome, we first calculated the GSVA HR pathway score for each tumor in the full BCBM RNA-Seq cohort (*N* = 45 patient-matched samples; Fig. 6e and Supplementary Fig. 10a, Supplementary Data 19). Consistent with the genomic analysis, high HR pathway scores were detected in BCBM relative to matched primary breast tumors in a detailed HR pathway analysis scoring for HR (*P* = 0.002), HRD230 (a 230 gene signature derived from HRD tumors)^26^ (*P* = 0.001), MMR (*P* = 0.001) and base excision repair (BER) (*P* < 0.0001) pathways (Fig. 6e and Supplementary Fig. 10B). In cases profiled for both RNA and DNA, we observe that majority of the Breast K mutational signature positive cases can also be detected using the RNA-based HR pathway analysis (Supplementary Fig. 10c). Of note, and similar to the mutational-based HRD methods, we do not observe an association with the enrichment of these pathways and diseases latency marked by brain metastasis-free survival (BMFS) or overall survival (OS) (Fig. 6e). However, the substantial enrichment in BCBM for molecular alterations, both at DNA and RNA level, impacting the HR pathway presents BCBM patients as potential candidates for PARP inhibitor therapy.

### HRD is functionally relevant in BCBM

**Fig. 7:**
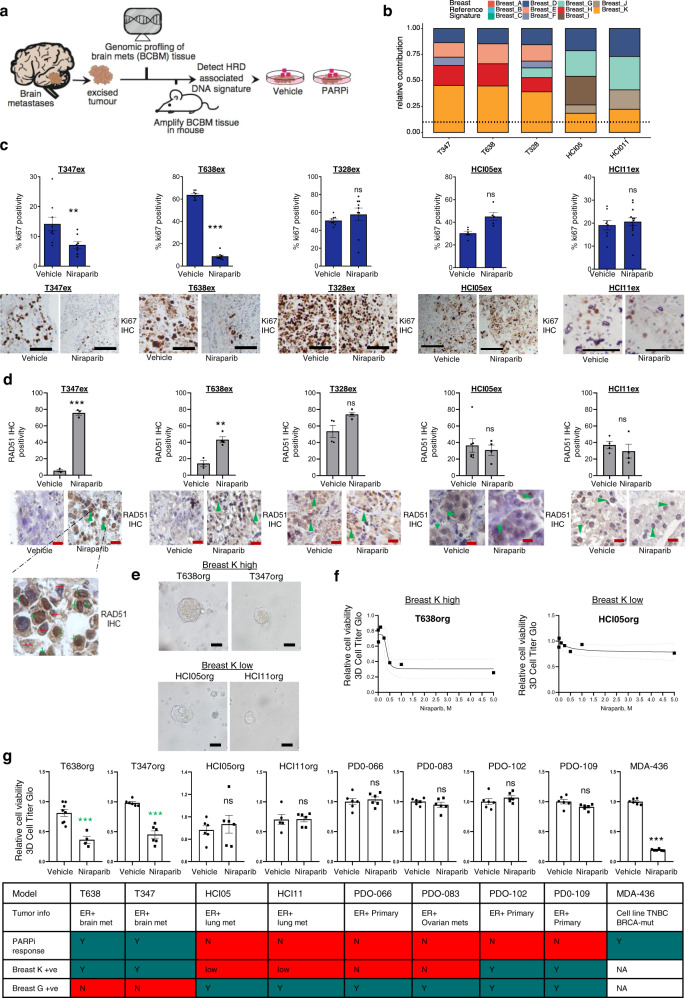
Ex vivo PARP inhibitor intervention study. **a** Schematic of ex vivo PARPi study. **b** Stacked bar chart of the relative contribution [0–1] of breast cancer-specific reference mutational signatures (Breast A-K) detected in each tumor explant. **c** PARPi demonstrates significant anti-tumor activity in patient-derived BCBM tumor explants (PDTEs). PDTEs were treated for 72 h with DMSO or 500 nM niraparib and ki67% (proliferation index) analyzed. Bar chart displays ki67% positivity (Representative ki67 images shown, Scale bars, 50 μm). Error bars represent mean ± s.e.m. (*n* = 3 biologically independent samples). Two-sided unpaired *t*-test with Welch’s correction (T347ex, *P* = 0.008; T638ex, *P* < 0.0001; T328ex, *P* = 0.38; HCI05ex, *P* = 0.01; HCI11ex, *P* = 0.588). **d** RAD51 IHC staining at 60x is displayed. Bar chart displays RAD51 nuclear positivity percentage. Zoomed in images of T347ex niraparib representative sample demonstrating RAD51+ve manual counts (+ve cells = green plus sign; –ve cells = red minus sign). (Black scale bars, 50μm; red scale bars 10 μm). IHC experiments were quantified from multiple tumor areas and at least 500 cells were assessed in each case. Two-sided unpaired *t*-tests with Welch’s correction (T347ex, *P* = 0.0002; T638ex, *P* = 0.003; T328ex, *P* = 0.06, HCI05ex, *P* = 0.59, HCI11ex, *P* = 0.48) (*n*  =  3 biologically independent samples). Error bars represent mean ± s.e.m. **e** Representative images of the fully established organoid cultures. Scale bars are 50 μm. (*n* = 4–8 biologically independent organoids). **f** A dose response curve for niraparib (0–5 µM). Each dot represents 4–8 replicates with the area of standard error illustrated by the dashed line. Cell viability is quantified by Cell Titer Glo 3D assay to measure ATP content. **g** Bar chart shows response to 500 nM niraparib for each of the organoid lines alongside key tumor characteristics and mutational signature content (Green fill color marks positive identification and red fill color marks negative identification). Error bars represent mean ± s.e.m. (*n* = 4–8 biologically independent organoids). Two-tailed unpaired *t*-test with Welch’s correction (T638org, *P* = 0.0006; T347org, *P* = 0.0002; T328ex, *P* = 0.06, HCI05org, *P* = 0.57, HCI11org, *P* = 0.94; PDO-066, *P* = 0.608; PDO-083, *P* = 0.32; PDO-102, *P* = 0.298; PDO-066, *P* = 0.075; PDO-066, *P* = 0.608; MDA-MB-436, *P* < 0.0001). Source data are provided as a Source Data file.

To understand the biological significance of this we further tested the functionality of the HRD in luminal BCBM using patient-derived tumor explants (PDTEs)^27^ and patient-derived organoid cultures (Supplementary Data 20). PDTEs were established from brain metastatic tissue from 3 breast cancer patients: T347 (ER^+^/HER2^−^ primary breast to ER^+^/HER2 amplified in BCBM), T638 (ER^+^/HER2^−^ primary breast tumor to ER^+^; gained HER2 expression in BCBM, *HER2* non-amplified), T328 (ER^+^/HER2^−^ in both primary breast and brain metastatic tumors) and from independent pleural/lung metastatic material in 2 of the samples HCI05 (ER^+^/HER2^+^) and HCI-011 (ER^+^/HER2^−^), all expanded in the mammary fat pad. WXS was performed on metastatic tumors for these patients, to identify somatic SNVs for mutational signature analysis using the Signal framework (Fig. 7a, Supplementary Data 20, see “Methods” section). In three BCBM models, we detected mutational signature Breast K (HRD), Breast E (analogous to RefSig), Breast D (MMR2), and Breast H (RefSig17;) alongside somatic *BRCA1/2* mutations of uncertain clinical significance. HCI05 and HCI11 harbored low Breast K and additionally Breast I (RefSig N1; *CTNNB1* driver mutation associated) and Breast J (RefSig 1) and no *BRCA1/2* mutations. Breast G (TP53 driver mutation associated) was detected in T328, HCI05, and HCI11 (Fig. 7b). PDTEs were treated for 72h in the presence or absence of PARP inhibitor (PARPi), niraparib, followed by IHC staining for ki67 cell proliferation marker. A significant anti-proliferative response to niraparib was observed in the T347 and T638 models (two-sided *t*-test; *P* < 0.01), but not in the T328, HCI05, and HCI11 models, commonly harboring Breast G, the *TP53* associated mutational signature (Fig. 7c). In addition, using the expression of RAD51, a core mediator of homologous recombination^28^, as an indicator of PARPi sensitivity, T347, and T638 models demonstrated low basal RAD51 (indicative of HR pathways defect and PARPi sensitivity) which elevated upon PARPi treatment. PDTEs T328, HCI05, and HCI11 models had strong RAD51 expression (HR proficient function, low/no sensitivity) were unaltered with treatment (Fig. 7d). We further extended our observations using organoid models of luminal breast cancer (Fig. 7e). We subjected the organoid lines to PARPi niraparib and assessed cell viability. We first verified all our explant experiments and demonstrated PARPi responses in T638 and T347 models (Breast K high and Breast G negative) and no response in HCI05 and HCI11 (lung/Pleural effusions; Breast K low/ Breast G high) (Fig. 7f, g). Furthermore, in patient-derived organoids from Breast K negative models, PDO-066 and PDO-083 (primary and ovarian metastasis); we observe no response to PARPi (Fig. 7g and Supplementary Fig. 11). Finally, we recapitulate the response observed in T328 in two models harboring Breast K high/ Breast G high profile (ie PD-102 and PDO-109). Similar to the T328 model, we see no response to PARPi (Fig. 7g). Therefore, understanding the relative contribution of specific mutational signatures in combination with RAD51 expression in *BRCA1/2/PALPB2* wild-type tumors may have significance in predicting response to PARPi in luminal BCBM.

## Discussion

Despite research efforts to decipher the intricacies of BCBM^6,10,12,13,15^, our understanding of brain metastatic disease especially in the context of individual clinical subtypes, has been remarkably limited. In this study, we have elucidated subtype-specific alterations in BCBM. Specifically, our data shows features of luminal BCBM leading to a complete remodeling of the BCBM transcriptomic and mutational landscape characterized by widespread alterations of HRD pathways.

Our results demonstrate unprecedented subtype-specific transcriptomic and genetic heterogeneity across a large cohort of BCBM patients, revealing biologically and potentially therapeutically significant pathways, alongside findings that will function as a critical reference to further advance the understanding of breast cancer brain metastases. While single-cell RNA-sequencing and multi-omics have been recently used for the profiling of the brain tumor microenvironment (TME)^8,29^ here, we employed a complementary approach using data-driven network analysis strategy in longitudinal patient samples revealing insight into dynamic BCBM gene programs. This approach presents evidence in support of metabolic reprogramming^30^ and dysregulation of immune response pathways^31^ for the HER2^+^ and TNBC subtype respectively. Notably, our findings also identify a brain-specific gene co-expression network in luminal BCBMs, enriched for cell cycle and *BRCA1*-mediated transcriptional regulation.

Previous studies have described *BRCA1/2*-mediated effects on the tumor in the context of both DNA damage repair deficiency and the tumor microenvironment^32^, while DNA repair deficiency has been reported in the context of brain metastases^33^ and BCBM^34,35^. Moreover, there is a reported association between *BRCA1/2* mutations and brain metastases in breast and ovarian cancer^36,37^. Our findings show DNA repair defect at both the DNA and RNA level. Strikingly, of the ~33% (13/39) of patients where we detect a mutational signature associated with HRD, >50% (6/11) were luminal. Within our BCBM samples where we find BREAST K signature enriched we observe that 75% of them are gained in BCBM compared to patient-matched primary. 8/13 samples have *TP53* mutations (not all of known functional significance) while we also see high (7/13) co-occurrence with *NF1* deletions. *NF1* mutations are associated with endocrine resistance^38^ which may partly explain the high co-occurrence in mostly luminal (endocrine-resistant) tumors. We found characteristic genomic imprints enriched in brain metastases, indicative of DNA repair deficiency corroborated by genomic scar scores and GSVA pathway activity. It is not yet clear whether the DNA-level HRD alterations are brain metastasis-specific alterations or general metastasis acquired traits as the current series did not contain patient-matched cases of extracranial tumors. Similarly, while we see no associations between mutational signature incidence and BCBM latency or treatment history, it is an important consideration given it has been reported that radiotherapy itself is associated with a ‘deletion signature’^22^.

The finding that BCBM tumors harbor high-frequency alterations in HRD pathways indicates that HRD brain metastatic tumors, in particular luminal subtypes, may benefit from a PARPi with intracranial activity^39,40^. HRD and PARPi sensitivity has previously been reported in the context of non-sporadic, familial, germline *BRCA1/2* mutated, and sporadic advanced breast cancer^41–43^.

Recently, results from the Phase II TBCRC-048 trial, have shown that PARP inhibition was effective for patients with germline *PALB2* and somatic *BRCA1/2* (independently of germline *BRCA* mutations)^44^. Consistent with the concept of BRCAness^45^, our findings here, define operative HRD in BCBM, independent of identifiable germline and/or somatic *BRCA1/2* mutations. Future studies will need to decipher the contribution of epigenetic silencing on HRD-associated signatures. In our expression analysis of BRCA1/2 and RAD51/c, we did not observe any significant evidence of expression loss in BCBM. However, *BRCA1* hypermethylation is known to confer a HRD and a transcriptional phenotype similar to TNBC tumors with *BRCA1*-inactivating variants. Additionally, epigenetic silencing of *RAD51C* and *BRCA1* by promoter methylation is also associated with Signature 3 (analogous to Breast K) and were shown to be highly enriched in TNBC^46^. Moreover, the number of samples with high Signature 3 that harbor epigenetic events in *BRCA1* and *RAD51C* is comparable to the number of germline events in *BRCA1/2*^46^.

We functionally validate our findings and report PARPi anti-tumor response in pre-clinical BCBM models harboring HRD mutational signatures. Interestingly, models enriched for the Breast G signature (RefSig30; *TP53* driver mutation associated) alongside the HRD signature were non-responders to PARPi. PARPi resistance in *TP53* mutated tumors has been reported^47,48^, however, further studies are needed to elucidate this association in the context of *TP53* driven mutational signature and its relationship with HRD-related signatures.

Work described here indicates that functionally relevant HRD signatures exist in BCBM independently of somatic and germline *BRCA1/2/PALB2* mutations and this presents an opportunity to extend the benefits of PARPi to a wider population of patients. In conclusion, this work opens further translational avenues for therapeutic interventions guided by subtype-specific HRD transcriptomic and genomic signatures and we believe these findings should inform future clinical studies.

## Methods

### Ethical issues

Institutional review boards from all three participating Institutions University of Pittsburgh, Royal College of Surgeons in Ireland and Mayo Clinic approved collection and analysis of specimens. For sequencing studies, the requirement for informed consent was waived by all three institutional review boards, considering all samples were de-identified, there was no more than minimal risk to human subjects, and all tissue was obtained as part of routine clinical care. Freshly resected breast cancer brain metastatic tumors utilized in tumor explant and organoid studies were collected with fully informed consent from patients and studied under approved IRB protocol #13/09/ICORG09/07 at the Royal College of Surgeons in Ireland. All procedures using animals were reviewed and approved by the Institutional Animal Care and Use Committee and the HPRA.

### Sample acquisition

The study population involves female breast cancer patients from three independent institutions, not pre-selected. A description of the covariate relevant study population and tumor characteristics including age, clinical tumor subtypes, pre- and post-menopausal status age groups, lines of treatment, and other clinical characteristics can be found in Supplementary Data 1. Patients had primary breast cancer and had subsequently developed brain metastasis. Only patients with FFPE tissue available for both primary breast and brain metastatic tumors were eligible to be included in the sequencing study. DNA/RNA was extracted from formalin-fixed paraffin-embedded (FFPE) tissue from patient-matched primary breast tumors and resected brain metastases using the Qiagen GeneRead DNA FFPE kit using standard protocols. Sample quality and concentration were assessed by Qubit and fragment analysis.

### Whole-exome DNA sequencing

#### Library preparation

For whole-exome sequencing (WXS), sheared DNA was processed using a SureSelect Human XT (low input) Human Exome v5 + UTR (v.5U) protocol (Agilent Technologies). Indexed, pooled libraries (4 per lane) were sequenced on an Illumina HiSeq4000 system (150-bp paired-end reads).

#### Sequence alignment and pre-processing

Sequencing reads were mapped to the human reference genome (hg19/GRCh37) using the Burrows-Wheeler Aligner (bwa mem v.0.7.13) using default parameters. According to the GATK4 best practice pipeline, read duplicates were marked using Picard (v.1.140). Sorted and de-duplicated alignments were next processed by base quality score recalibration (BQSR).

#### Brastianos et al.^12^ WXS BCBM Cohort

Whole-exome sequencing data for 21 breast cancer brain metastases cases (63 trios of matched normal (buffy coat plasma-derived germline), primary breast and brain metastatic tumor) from the Brastianos et al.^12^ study were downloaded from the database of Genotypes and Phenotypes (dbGap) (accession number phs000730.v1.pl)^12^. Sequencing reads were aligned to human reference genome hg19 using bwa mem v.0.7.13, with post-processing of sequence alignment files according to GATK4 best practice pipeline^49^.

### Allele-specific DNA copy number inference

Total and allele-specific copy number states were inferred for all tumor samples using FACETS Suite (v2.0.8) and FACETS (v.0.6.1) (https://github.com/mskcc/facets-suite). Tumor and matched normal bam files were pre-processed using snp-pileup (v.0.6.1) with parameters –q15 –Q20 –P100 –r25,0. A two-pass implementation of FACETS using snp-pileup files as input, was utilized were a low sensitivity run (cval = 150) first infers the purity and log-ratio related to diploidy, as per^50^ methodology. A second higher sensitivity run (cval = 25) to detect focal events, determines the copy number state of each gene.

### Calculation of genomic scar scores

Genomic instability can be measured by genomic scar scores i.e., unique fingerprints embedded in tumor samples from copy number alteration profiles. For homologous recombination deficient (HRD) tumors, the copy number alteration profile is distinct, marred by characteristics that can distinguish them from HR proficient tumors: three genomic scar scores: HRD loss of heterozygosity (HRD-LOH), large state transitions (LST), and number of telomeric allelic imbalance (ntAI), each an independent marker of chromosomal and genomic instability associated with HRD. The three genomic scar scores were calculated from allele-specific copy number calls in FACETS: (1) fraction of chromosome which contains loss of heterozygosity (LOH), (2) Large state transitions (LST), (3) Number telomeric allele imbalance (ntAI) events. Combined genomic scar score was calculated as per Telli et al.^25^ HR deficiency was defined as high HRD score (above the HRD threshold, > 42). HRD score was defined as the unweighted sum of LOH, TAI, and LST scores: HRD = LOH + TAI + LST. Details of the individual LOH, TAI, and LST scores, as well as the combined HRD score, are described in Supplementary Data 17.

### Identification of recurrent somatic copy number alterations

Segmentation files from FACETS allele-specific copy number calling were used as input for identification of recurrent amplifications and deletions using GISTIC2.0 (version 2.0.23) (https://github.com/broadinstitute/gistic2)^51^. GISTIC2 was run separately on the primary breast tumors (*N* = 39 samples) and brain metastatic tumors (*N* = 39 samples) in order to identify recurrent SCNA specific to disease status. GISTIC2.0 parameters used were amplification and deletion thresholds (ta,td) = 0.1; qvt < 0.25; maxseg 4000; brlen(broad length cutoff) = 0.5; confidence level of 90%; genegistic 1; armpeel 1. GISTIC2.0 outputs both significant broad (arm) level and focal regions of significant SCNA. Significant broad arm level alterations were defined as follows. High-level amplifications >6 copies, gain >2 copies; loss is >copy loss and deletion >2 copy homozygous deletion. Focal SCNA are labeled as −2,−1,0,1,2 where −2 refers to homozygous deletions, 2 refers to high-level amplifications, −1 hemizygous i.e., gene loss, with 1 referring to copy number gain and 0: no SCNA.

### Somatic mutation calling

Somatic single nucleotide variants (SNVs), insertions, and deletions (indels) were called using Mutect2 (v.4.1.2)^49^ and Strelka^52^ (v. 2.9.8) respectively from matched normal and tumor pairs. In order to filter for false-positive somatic mutation calls, Mutect2 and Strelka calls were filtered against a panel of normal (PON) samples, generated using the CreateSomaticPanelOfNormals function part of the GATK4 best practice pipeline. As the *N* = 18 and *N* = 21 WXS BCBM cases were generated from different library preparation methods, sequencing technology, and centres, we generated a PON separately for the *N* = 18 and *N* = 21 normal tissues. FFPE samples are known to contain mutational biases in the C > T/G > A transition. OxoG filter was applied through the read orientation bias model with Mutect2 to remove mutations with FFPE strand bias. Bcftools [http://samtools.github.io/bcftools/bcftools.html] *norm* function was used to left align and normalize indels. Additional filtering was applied for FFPE false-positive calls using the ffpe-filter of ngs filter [https://github.com/mskcc/ngs-filters], with variants also filtered according to germline variants reported in ExAC at a population minor allele frequency > 0.05. Variants passing quality control were annotated using MSK vcf2maf [https://github.com/mskcc/vcf2maf] and variant effect predictor (VEP) using GRCh37, which outputs both a.vcf and.maf file format. Annotated maf files were used by MAFTools^53^ for downstream somatic mutation analysis, with annotated.vcf used as input for mutational signature analysis. Cancer cell fraction (CCF) of mutations were calculated using FACETS Suite based on the McGranahan et al. methodology^54^.

### Identification of driver mutations

dNdScv was used to analyze annotated somatic SNVs and indels for evidence of positive selection based on mutation frequency above background rate (the ratio of non-synonymous to synonymous mutations (d*N*/d*S*))^55^. Driver mutations were detected using the dndscv R package with default parameters: using a Poisson-based d*N*/d*S* model (under the full trinucleotide context model 192 rate substitution model); max_coding_muts_per_sample = 3000 (hypermutator samples are removed to improve driver mutation sensitivity) (https://github.com/im3sanger/dndscv). Statistically significant driver genes were called based on a global *q*-value < 0.1.

### Estimation of tumor mutational burden

Tumor mutational burden (TMB) is defined here as the number of somatic mutations per megabase of exome. The mutation rate per Mb was calculated using maftools as the total number of coding variants (SNVs, indels) divided by the length of the capture in megabases (50 Mb).

### Data sets for BCBM associated genomic alterations

Focal somatic copy number alterations and statistically significant somatic driver mutations identified using dNdScv (*q*-value < 0.1) were cross-referenced to previously reported breast cancer brain metastatic genomic alterations^12,56^. Along with genomic alterations in BCBM reported in the Brastianos et al. study^12^, Supplementary Table 4 was downloaded from the Rinaldi et al.^56^ targeted sequencing study of approx. 11,000 unmatched primary breast, local recurrence and distant metastatic tumors using the FoundationOne assay. Supplementary Table 4 details genomic alterations enriched by site of metastases, including 238 brain metastatic tumors, relative to primary breast tumor and local recurrence alteration frequency. coMut python library was used to visualize co-occurrence and frequency of SCNA and SNVs in brain metastatic tumors^57^.

### Germline mutation calling

Germline mutation calling was performed for the DNA repair genes, BRCA1, BRCA2 and PALB2, using GATK HaplotypeCaller (v. 4.1.2), in GVCF mode, from germline normal sample BAM files. Germline variants were filtered using the VariantFiltration function by applying the following cutoffs to (a) SNPs: QD < 2.0; FS > 60.0; MQ < 40.0; MQRankSum < −12.5; ReadPosRankSum < −8.0; SOR > 3.0 and (b) INDELS: QD < 2.0; FS > 200.0; ReadPosRankSum < −20.0; SOR > 10.0. Germline variants which passed quality based filtering were extracted using GATK SelectVariants, followed by annotation using Variant Effect Predictor (VEP) GRCh37, prioritized based on described clinical significance and pathogenicity in the NCBI ClinVar Database and IMPACT annotation. Only those variants annotated as ClinVar annotation predicted: *“likely pathogenic”, “pathogenic”* or *“variant of uncertain significance (VUS)”* were reported.

### Mutational signatures

Somatic point mutations from matched normal-tumor mutation calling were used for mutational signature analysis. Signal^23^ [https://signal.mutationalsignatures.com/analyse] a framework for organ-specific mutational signature analysis was used with the following parameters: non-PASS variants filtered out, GRCh37 human genome reference. For SignatureFit algorithm: breast originating organ, number of bootstraps 100, threshold *k* = 5, *P*-value < 0.05. Somatic single base substitutions are categorized by their trinucleotide context to generate a 96-channel mutational profile. Regions of clustered substitutions i.e., kaetegis regions were filtered. Extraction of mutational signatures from somatic mutation catalogs in cancer was performed using the Signal framework optimal mutational signature extraction algorithm. Fitted signatures were compared to organ-specific mutational profiles in the Signal database using cosine similarity measure. The SignatureFit algorithm determines the relative contribution of each signature by bootstrapping (*n* = 100 iterations) the tumor somatic mutation catalog (vcf), generating multiple SignatureFit solutions in order to estimate the empirical probability distribution of an exposure to be larger or equal to a given threshold (i.e., 5% of mutations of a sample). From bootstrapped solutions, a point estimate of the mutation count for each signature is extracted, where the point estimate is the median of the distribution of counts for a candidate signature. Those candidate mutational signatures with a point estimate below a threshold (5% of the total number of mutations in the sample), will have signature point estimates set to 0. In text, when describing the organ-specific signatures Breast A-K, reference signatures are also annotated according to ref. ^23^. Reference signatures were numbered according to the most similar COSMIC substitution signature when possible without ambiguity. For instance, RefSig 1 is equivalent to COSMIC signature 1 (v3.1).

### Exome capture RNA sequencing

#### Library preparation

Library preparation for RNA-seq was performed using 100 ng of total RNA and a TruSeq Stranded Total RNA (Degraded RNA) v2 RNA Exome Library and TS RNA Access capture protocol (Illumina). Indexed, pooled libraries (3 per lane) were sequenced on an Illumina HiSeq4000 system (100 bp paired-end reads). Details of sample acquisition, tissue processing, and RNA-sequencing library preparation for patient-matched primary breast and brain metastatic tumor samples for *N* = 21/ 45 patients (*N* = 42 samples; PITT-RCSI Cohort) are detailed here^15^.

### Exome capture RNA sequencing data processing

FastQC was used to assess quality control metrics for paired-end sequencing reads (FASTQ) for all 90 samples. For PITT-RCSI FASTQ, if fastQC flags indicated adapter contamination and/or poor quality base calls, BBDuk (version 38) from the BBMap toolkit was used for Illumina sequencing adapter removal and read trimming using the following parameters: *minlen* = *50, qtrim* = *rl, trimq* = *10, ktrim* = *r, k* = *25, mink* = *11, hdist* = *1, tpe tbo*. Salmon (v.0.91) was used to perform quasi-mapping of sequencing reads, with *seqBias* and *gcBias* corrections enabled, using a 31bp k-mer index of the GRCh38.p10 (GENCODE v.27) human reference transcripts, to estimate transcript abundance for each sample. In order to quantify comprehensive mapping rates and other quality control metrics, adjunct to Salmon read mapping, two-pass read alignment was performed using STAR (v2.6.1a), followed by RSeQC and MultiQC for visualization and assessment.

### Gene expression quantification

Tximport package was used to import transcript abundance estimates from *quant.sf* files, generated by Salmon read mapping into R statistical programming environment for gene expression quantification. Transcript abundance estimates were collapsed to gene-level gene expression counts. TXI data objects for MAYO and PITT-RCSI RNA-Seq cohort, containing unprocessed Salmon read counts, transcript per million (TPM), and gene length values were combined for subsequent downstream analysis. Gene filtering, normalization and batch correction methods are fully described in Supplementary Information.

### Unsupervised hierarchical clustering

For evaluation of potential batch-driven effect, unsupervised hierarchical clustering was performed using the *hclust* function in R. A matrix of sample-to-sample Euclidean distance values was calculated from log2 variance stabilized transformed (VST) gene expression counts using the *dist* function. The *ward* D2 linkage algorithm was used for sample clustering. Sample clustering was visualized as a dendrogram using *plotDendroAndColors()* function from WGCNA R package, with the sample tree annotated with clinicopathological variables: disease status (primary breast or brain metastases*)*, sequencing batch (#1–5), ER status by IHC (ER^+/−^), IHC subtype (ER^+^/HER2^−^, HER2^+^, TNBC and histological subtype (IDC, ILC, Other)).

### Intrinsic molecular subtyping using PAM50

Prior to subtype classification, test set bias due to proportion of ER^+^ to ER^−^ tumor class imbalance was assessed^58^. The proportion of ER^+^ to ER^−^ tumor subtypes for all 90 samples were ~43 to 57%. The intrinsic molecular subtype of each tumor was called using the genefu R package^59^, by applying the Parker et al.^60^ PAM50 subtype predictor to gene expression data. Batch corrected log2 normalized counts (log2 CPM TMM) were used as input to the *molecular.subtyping()* function, setting a seed prior to classification for reproducibility. Discrete subtype assignment (LumA/LumB/Her2-E/Basal/Normal) for each tumor was made based on the max probability score, calculated from Spearman correlation of gene expression profile to its closest centroid. A confusion matrix showed 98% (41/42) of samples that had a previously predicted subtype call for 21/45 cases in the RNA-Seq Cohort^15^, agreed with PAM50 subtype classification performed here. Note, on manual inspection of PAM50 subtype calls, sample MAYO_BM_11 was changed from Basal to Her2 PAM50 subtype, based on the IHC subtype call and probability score which was borderline. Subtype distribution across primary and brain metastatic tumors was plotted using *ggpubr*, with Sankey diagram generated using SankeyMATIC to visualize intrinsic molecular subtype switching, with labeling added using Adobe Illustrator.

### Subtype-specific differential gene expression

For subtype-specific differential gene expression (DGE) testing, patients were first stratified based on the IHC subtype of their primary tumor: ER^+^/HER2^−^ (Luminal); HER2^+^; Triple-negative breast cancer (TNBC). For each patient/IHC subtype group, differential gene expression testing was carried out using DESeq2^61^, comparing brain metastatic (BM) tumor to primary breast tumor, using the following formulae for the design matrix: *~SV1+ patientID + tumourID (BCBM vs Primary)*, where SV1 is a coefficient weight vector included in the model to adjust for batch driven effect. Non-negative, filtered, un-normalized protein coding gene expression integer value counts from Salmon were used as input to DESeq2. The statistical distribution used to model RNA-Seq count data (characterized by overdispersion: variance > mean) is the negative binomial distribution. The DESeq2 negative binomial model corrects counts for sequencing library size. A gene was defined as differentially expressed based on a Benjamini & Hochberg adjusted *P*-value < 0.05 (Wald test) and a log2 Fold Change (FC) ± 2.0.

### DGE clustering and heatmap

For each subtype-specific comparison, unsupervised hierarchical clustering and heatmap visualization was performed using *ComplexHeatmap* in R^62^. Genes identified as differentially expressed were clustered using the ward D2 linkage method, based on the (1-Pearson correlation coefficient) dissimilarity distance metric, with samples clustered based on Euclidean distance metric. In order to split the gene clustering dendrogram generated by *Heatmap* function, genes were first clustered using the partitioning around medoids (PAM)/*k-*medoids method, as part of the *cluster* R package, in which each gene was assigned to a cluster with the nearest medoid. In this method, each cluster is represented by a medoid, which is a gene that corresponds to the most centrally located point within the gene expression cluster as a whole. In order to objectively select the number of clusters *k* for PAM, the *NbClust function in R* was used with the following parameters: *min.nc* = *2, max.nc* = *10, distance* = *“euclidean”, method* = *“kmeans”*.

### Weighted gene co-expression network analysis (WGCNA)

The WGCNA method^63^ was used to identify subtype-specific gene co-expression networks separately for primary breast and brain metastatic tumors. Batch corrected log2 variance stabilized transformed (VST) gene expression counts, filtered by TPM, were used for correlation network analysis. Full details in addition to gene module preservation analysis and differential gene co-expression network analysis are provided in the Supplementary Information file.

### Network union and visualization

For each molecular subtype-specific analysis, the network containing preserved gene modules was assigned Graph *G*_1_, with the network containing differential co-expression network modules assigned Graph *G*_*2*_. The *igraph* R *graph.union()* function was used to generate the union of Graph *G*_1_ and *G*_*2*_ which represents the gene network that contains both preserved and enriched gene co-expression network modules in breast cancer brain metastases. The network degree statistic was calculated using *igraph degree()* function. For network visualization, the *ggnetwork* (https://briatte.github.io/ggnetwork/) and *viridis (*https://github.com/sjmgarnier/viridis*)* R package were used.

### Gene set enrichment analysis (GSEA)

To identify functional processes and pathways significantly enriched or depleted in brain metastases compared to primary breast tumors, gene set enrichment analysis (GSEA) was applied separately to each k-medoid cluster (Cluster 1,2) identified from subtype-specific significantly differentially expressed genes. Genes in each cluster were ranked according to median gene expression z-score in brain metastatic tumor samples. GSEA was also performed on gene modules identified from network analysis, where genes were pre-ranked based on log2 fold change values from DGE. For GSEA, *fgsea* R package was used with molecular signature database (MSigDB v.6.2) and the following gene sets: hallmarks, curated (C2), cancer orientated (C4), oncogenic signatures derived from gene perturbation studies (C6), immune-related signatures (C7), KEGG pathways, Gene Ontology (BP, MF pathways). *fgsea* was run with these parameters: *minSize = 5, maxSize* = *500, number of permutations* = *10,000*. Significantly enriched pathways were defined based on an FDR < 0.25 and absolute normalized enrichment score (NES) > 1.0. Cytoscape (v.3.7) *EnrichmentMap* plugin was used to visualize statistically significant pathways for each subtype from GSEA of network gene modules (FDR < 0.01; NES ± 1.0).

### Breast cancer metastases gene expression data sets

#### Siegel et al.^14^ RNA-Seq Cohort

FASTQ files for previously published total RNA-Seq data of patient-matched primary breast with multi-organ metastatic tumor (*N* = 16 patients; 68 metastases) were downloaded from the NCBI’s genotypes and phenotypes database (dbGaP) (accession number phs000676)^14^. Paired-end sequencing reads were processed using the same methodology for MAYO-PITT-RCSI Cohort above.

#### Microarray data

Microarray-derived RMA normalized gene expression matrices of multi-organ breast metastatic tumors (GSE14017^64^, GSE14018^64^) and GSE14018 generated on the Affymetrix HGU133plus2 and HGU133A chips, respectively, were downloaded from Gene Expression Omnibus (GEO) using the *GEOquery* R package. For each gene profiled, the probe with the greatest variability (IQR) across samples was selected using the *genefilter::findLargest*() function in R. Probe IDS were mapped to gene symbol using *biomaRt*^65^ and the Affymetrix HGU133plus2 and HUG133A probe annotation databases.

### Single sample GSEA (ssGSEA) of gene modules

For each subtype-specific gene network module, normalized gene expression values from publicly available, independent, multi-organ breast cancer metastases data sets, were used to calculate a single sample gene set enrichment score (ssGSEA) using the *gsva()* function (*method = ‘ssgsea’*) apart of the *GSVA* R package. The Wilcoxon Rank-Sum test was used to test if ssGSEA score for each gene module was significantly different (adjusted *P*-value < 0.05) in brain metastases versus all other metastatic tumor scores. The *ggplot2 geom tile_plot() was* used to visualize results.

### DNA repair pathway gene sets

DNA repair pathway gene sets downloaded from KEGG database using the MSigDB gene signature and pathway repository (v.6.2) (https://www.gsea-msigdb.org/gsea/msigdb) were: homologous recombination (HR), mismatch repair (MMR), base excision repair (BER), non-homologous end joining (NHEJ). A 230 member gene signature associated with homologous recombination deficiency (HRD230) was obtained from^26^. Network genes were cross-referenced against genes in the *“DNA Repair”* category of the Drug-Gene Interaction database (https://www.dgidb.org/) version 3.0 (DGIdb 3.0).

### Gene set variation analysis (GSVA)

Batch corrected log2 normalized counts (TPM) were used to calculate GSVA scores for DNA repair pathway gene sets for each sample in the RNA-Seq BCBM cohort (*N* = 90 samples), using the *GSVA* R package. GSVA normalized enrichment scores [−1,1] represent the relative enrichment of a gene set in each sample relative to all other tumors of the analyzed cohort. A paired Wilcoxon signed-rank test (*P*-value < 0.05) was used to compare GSVA pathway score in patient-matched brain metastatic vs primary breast tumor for each gene set. GSVA scores were plotted using the *ggpubr* function *ggpaired*() for boxplots and/or as heatmap using the *ComplexHeatmap* R package.

### Patient-derived tumor explant models

Tumor tissues were processed under sterile conditions and tumor fragments were implanted into the mammary fat pad of female NOD-SCID (NOD.CB17-Prkdc<scid>/NcrCrl) (mice (*N* = 5)) to establish patient-derived xenografts and amplify the brain metastatic tissue^66,67^. ER^+^ tumors were supplemented with estradiol. When tumors reached 1.5 cm in diameter they were harvested and viably biobanked. HCI05 and HCI-011 models were a kind gift from Alana Welm lab^67^. Patient-derived tumor explant (PDTEs) of luminal brain metastasis (T347, T638 and T328) were established by culturing 2–4 mm^3^ biobanked tumor fragments on hemostatic gelatin dental sponges (Vetspon, Novartis) pre-soaked with human mammary epithelial media as described previously^27^. The PDTEs were treated with Niraparib or DMSO for 72 h after which they were paraffin-embedded and profiled with immunohistochemistry (IHC). Niraparib treatment concentration of 500 nM was selected representing approximately the peak plasma concentration measured in patients receiving a daily oral dose of 300 mg^68^. IHC for RAD51 (1:200; mouse monoclonal, Genetex, GTX70230) and ki67 (1:50 MIB-1 clone, Dako, M7240) was carried out using a Dako EnVisionTM Kit with antigen retrieval carried out as per manufacturer’s instructions. Positivity scores were assessed and scored utilizing Aperio ImageScope software using the positive pixel algorithm. The viability of the tumors was evaluated by utilizing ki67 as a proliferation marker to identify proliferating cells.

### Patient-derived tumor organoids

Organoids were established from tumors collected and processed under IRB approval from both participating institutions University of Pittsburgh and the Royal College of Surgeons in Ireland. Organoid lines were generated from tumors following Sachs et al.’s protocol^69^ with the addition of estradiol supplementation for ER+ tumors. Established organoids were dissociated into single cells and seeded in organoid media with 5% of Cultrex® Reduced Growth Factor Basement Membrane Matrix, type 2 (BME, Trevigen, 3533-001-02) for the intervention experiment. 24hrs after seeding, organoids were treated with vehicle or niraparib (*N* = 4–8). Cell viability was measured 7 days post-treatment using CellTiter-Glo® 3D Cell Viability assay (Promega). MDA-MB-436 (ATCC) cells were utilized as positive control. Cells used were authenticated (SourceBioScience) and regularly tested for mycoplasma contamination (LT07-118, Lonza).

#### WXS sequencing

DNA was extracted from tumors using the Qiagen GeneRead DNA FFPE kit using standard protocols. Sheared gDNA was processed using the KAPA library preparation kits, and subsequently, the libraries were captured using Agilent SureSelect Human All Exon v.5 (Agilent Technologies). Sequencing was carried out using the BGISEQ sequencing system followed by initial data pre-processing by BGI Genomics (Hong Kong). HCI tumors used to establish the PDXs and organoid lines were WES profiled using the Agilent SureSelectXT Human All Exon V6+COSMIC or Agilent Human All Exon 50 Mb library preparation protocol Sequencing was carried out on Illumina HiSeq 2500 instrument. Paired-end sequencing reads (FASTQ file format) were aligned to the hg19 reference human genome using BWA read alignment. Aligned sequenced reads were pre-processed using the best practise GATK pipeline. Single nucleotide variants (SNVs) were called using Mutect2 using tumor-only mode (no matched normal sample) (v.4.1.2)^49^. SNVs were filtered against a previously generated panel of normal (PON) followed by previously described variant filtering steps and annotation

### Statistics and reproducibility

Statistical analyses were performed using the base stats R package. Reported *q* values represent Benjamini–Hochberg corrected *P*-values. All statistical tests (paired Wilcoxon Rank-sum (Mann–Whitney *U*-test), Student’s *t*-test etc) were two-sided unless otherwise stated. No statistical method was used to predetermine sample size. The investigators were blinded for immunohistochemical analyses.

### Reporting summary

Further information on research design is available in the Nature Research Reporting Summary linked to this article.

## Supplementary information

SUPPLEMENTARY INFO

Description of the Additional Supplementary Files

Supplementary Data 1

Supplementary Data 2

Supplementary Data 3

Supplementary Data 4

Supplementary Data 5

Supplementary Data 6

Supplementary Data 7

Supplementary Data 8

Supplementary Data 9

Supplementary Data 10

Supplementary Data 11

Supplementary Data 12

Supplementary Data 13

Supplementary Data 14

Supplementary Data 15

Supplementary Data 16

Supplementary Data 17

Supplementary Data 18

Supplementary Data 19

Supplementary Data 20

Reporting Summary

## Data Availability

In line with Institutional Review Board approvals from all three participating Institutions including the University of Pittsburgh, Royal College of Surgeons in Ireland, and Mayo Clinic, raw RNA (*N* = 45 patients/*N* = 90 breast cancer brain metastasis cases) and WES DNA (*N* = 18 matched normal, primary breast and brain metastatic tumor) data was not deposited in a public repository as informed consent was not available with these samples. Raw RNA and DNA sequencing data for the paired primary and metastatic samples will be made available upon request and under regulatory compliance via data usage agreement (DUA). Please contact the corresponding author with data access requests that will be granted once the DUA is signed. Processed RNA-sequencing data for all cases reported in the study (*N* = 45 patients/*N* = 90 breast cancer brain metastasis cases) is deposited in the Gene Expression Omnibus under the accession number GSE184869. For the WES DNA (*N* = 18 matched normal, primary breast and brain metastatic tumor) samples newly generated as part of the study, the processed files are available on figshare [10.6084/m9.figshare.16685680.v1]. WES data for 21 of the 39 breast cancer brain metastases cases (matched normal, primary breast, and brain metastatic tumor) has been described previously and are available to download upon request from the database of Genotypes and Phenotypes (dbGap) (accession number phs000730.v1.pl). RNA-Seq data from Siegel et al.^14^ (*N* = 16 patients; 68 metastases) were downloaded from the dbGaP (accession number phs000676). Supplementary Table 4 from the Rinaldi et al.^56^ targeted sequencing study of approx. 11,000 unmatched primary breast, local recurrence, and distant metastatic tumors using the FoundationOne assay is available at 10.1371/journal.pone.0231999. For GSEA the molecular signature database (MSigDB v.6.2) is available at https://www.gsea-msigdb.org/gsea/msigdb. The 230 member gene signature associated with homologous recombination deficiency (HRD230) was obtained from https://www.nature.com/articles/ncomms4361#Sec22. Network genes were cross-referenced against genes in the *“DNA Repair”* category of the Drug-Gene Interaction database [https://www.dgidb.org/] version 3.0 (DGIdb 3.0). The microarray-derived gene expression data for the multi-organ breast metastatic tumors is available for download on GEO using the accession IDs: GSE14017 and GSE14018. Source data are provided with this paper.

## Source data

SOURCE DATA
