# Supplementary material for: Mapping molecular subtype specific alterations in breast cancer brain metastases identifies clinically relevant vulnerabilities
Source: Nat Commun. 2022 Jan 26;13:514. doi: 10.1038/s41467-022-27987-5 (PMC8791982; doi:10.1038/s41467-022-27987-5)
Supplement: Supplementary file 1 — SUPPLEMENTARY INFO [file 41467_2022_27987_MOESM1_ESM.pdf]

Supplementary Information for

Mapping molecular subtype specific alterations in breast cancer brain metastases identifies clinically relevant vulnerabilities

Nicola Cosgrove[1#], Damir Varešlija[1#], Stephen Keelan[1], Ashuvinee Elangovan[7], Jennifer M Atkinson[7], Sinéad Cocchiglia[1], Fiona T. Bane[1], Vikrant Singh[1],Simon Furney[2], Chunling Hu[3], Jodi M Carter[3], Steven N Hart[4], Siddhartha Yadav[5], Matthew P Goetz[5], Arnold D.K. Hill[1], Steffi Oesterreich[7], Adrian V Lee[8], Fergus J Couch[3]* and Leonie S Young[1]*

These authors contributed equally: Nicola Cosgrove, Damir Varešlija

These authors jointly supervised this work: Fergus J Couch, Leonie S Young

*email: couch.fergus@mayo.edu and lyoung@rcsi.ie

The PDF file includes:

Supplementary Figures 1
Supplementary Figure 1: Overview of Exome Capture RNA-Seq and quality control. 1
Supplementary Figure 2: Batch effect assessment. 2
Supplementary Figure 3: Results from testing for the relationship between SV1 and batch effect in comparison to clinically relevant tumour characteristics using linear model. 3
Supplementary Figure 4: Method summary for gene co-expression network analysis in brain metastases. 4
Supplementary Figure 5: Summary of results from weighted gene co-expression network analysis (WGCNA). 5
Supplementary Figure 6: Subtype specific differential gene co-expression networks in BCBM. 6
Supplementary Figure 7: Gene network module ranking by independent single sample enrichment testing in previously published breast cancer metastases gene expression data 7
Supplementary Figure 8: Co-expression module genes categorized by both subtype and DgIdb gene categories 8
Supplementary Figure 9: Mutational signature profile all tumors. 9
Supplementary Figure 10: HRD related signatures detected with RNA profiling. 10
Supplementary Figure 11: Niraparib response in patient-derived organoids 11

Supplementary Methods 12
Gene Filtering and Normalisation 12
Principal Component Analysis 12
Batch effect correction 12
Weighted gene co-expression network analysis (WGCNA) 13
Gene module preservation analysis 14
Differential gene co-expression network analysis 15

Supplementary References 16

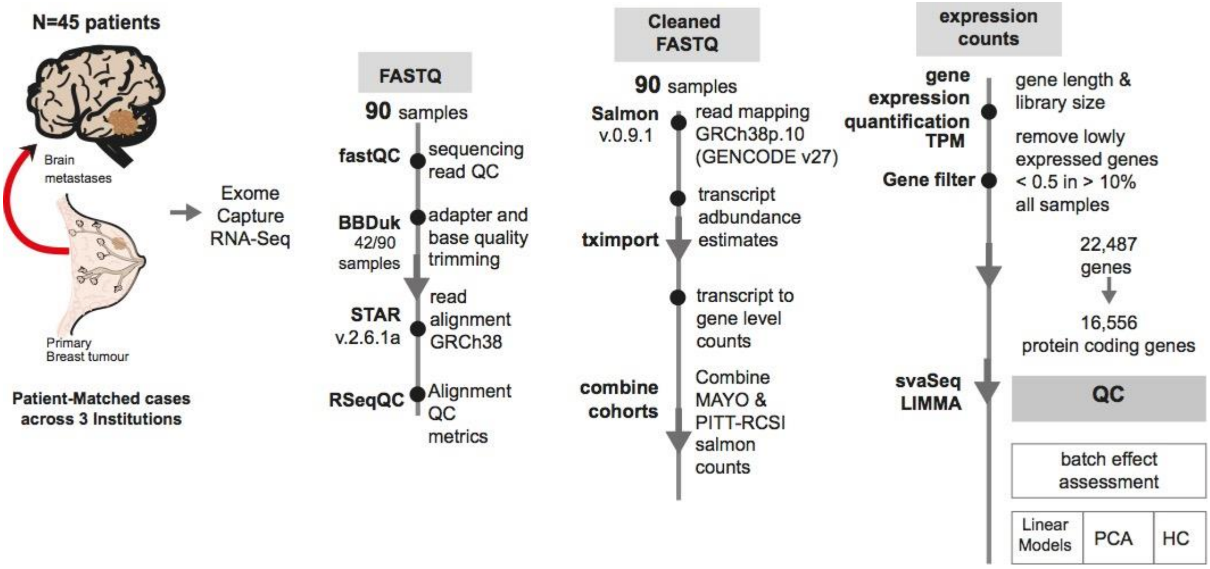

Supplementary Figure 1: Overview of Exome Capture RNA-Seq and quality control.
Exome Capture RNA Seq cohort contains patient matched primary breast tumour and brain metastatic tumour samples for 45 patients (N=90 samples), gathered from three institutions. Sequencing reads (FASTQ) went through initial quality control, followed by read alignment using STAR for comprehensive assessment of data quality. For gene expression studies, cleaned FASTQ post initial QC were mapped to human reference transcripts GRCh38p.10 using Salmon, followed by gene expression quantification, gene filtering using TPM and selection of protein coding genes. Batch effect assessment was carried out as sequencing was performed in different facilities and on different dates, using svaSeq, LIMMA, linear modelling, PCA and hierarchical clustering (HC).

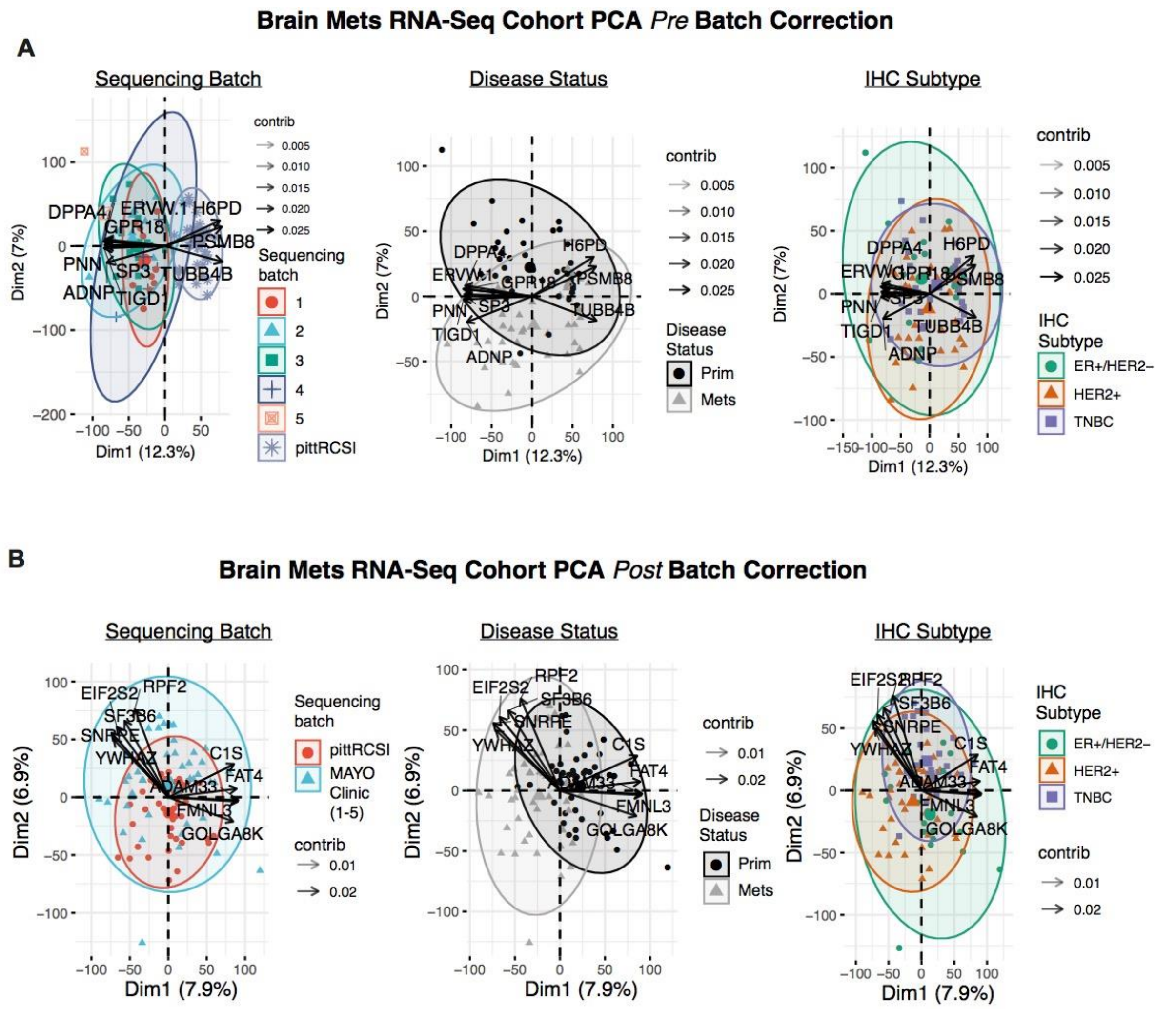

Supplementary Figure 2: Batch effect assessment.

(a) Biplots of Principal component analysis (PCA), capturing the largest sources of variation across all 90 samples, pre batch effect correction of gene expression data. Biplots show variation captured by Dim1 vs Dim2 for, from Left-Right, sequencing batch (MAYO batches #1-5, PITT-RCSI batch), Disease Status (Primary breast tumour (Prim), brain metastatic tumour (Mets)), IHC tumour subtype (ER+/HER2-,HER2+,TNBC). **(b)** Biplots of Principal component analysis (PCA), capturing the largest sources of variation across all 90 samples, post batch effect correction of gene expression data.

	SV1 (Dependent variable)				
	Batch	*Tumour*	*IHC*	*Histology*	*All*
**Batch (Centre)**					
pittRCSI	-0.094***				
MAYO	0.083***				0.175***
**Tumour**					
Prim		0.004			
Mets		-0.004			-0.01
**IHC Subtype**					
ER+/HER2-			0.028		
HER2+			0.014		0.004
TNBC			-0.035*		-0.039**
**Histological Subtype**					
IDC				-0.003	
ILC				-0.021	-0.028
Other				0.041	0.033
**Constant**					-0.078***
Observations	90	90	90	90	90
R2	**0.701**	**0.002**	**0.062**	**0.016**	**0.755**
Adjusted R2	**0.694**	**-0.021**	**0.03**	**-0.018**	**0.738**
Residual Std. Error	0.058 (df = 88)	0.107 (df = 88	0.104 (df = 87)	0.106 (df = 87)	0.054 (df = 83)
F Statistic	102.954*** (df = 2; 88)	0.08 (df = 2; 88)	1.912 (df = 3; 87)	0.47 (df = 3; 87)	42.720*** (df = 6; 83)
Note:	*p<0.1;	**p<0.05;	***p<0.01		

Supplementary Figure 3: Results from testing for the relationship between SV1 and batch effect in comparison to clinically relevant tumour characteristics using linear model. Highlighted in orange is the significant association between SV1 and batch (F-statistic 102.954 on 2 and 88 degrees of freedom (df), p < 0.01), with an *R2* of .701 (+/- 0.058) i.e. ~0.70% of the total variance (0.76%) explained by centre.

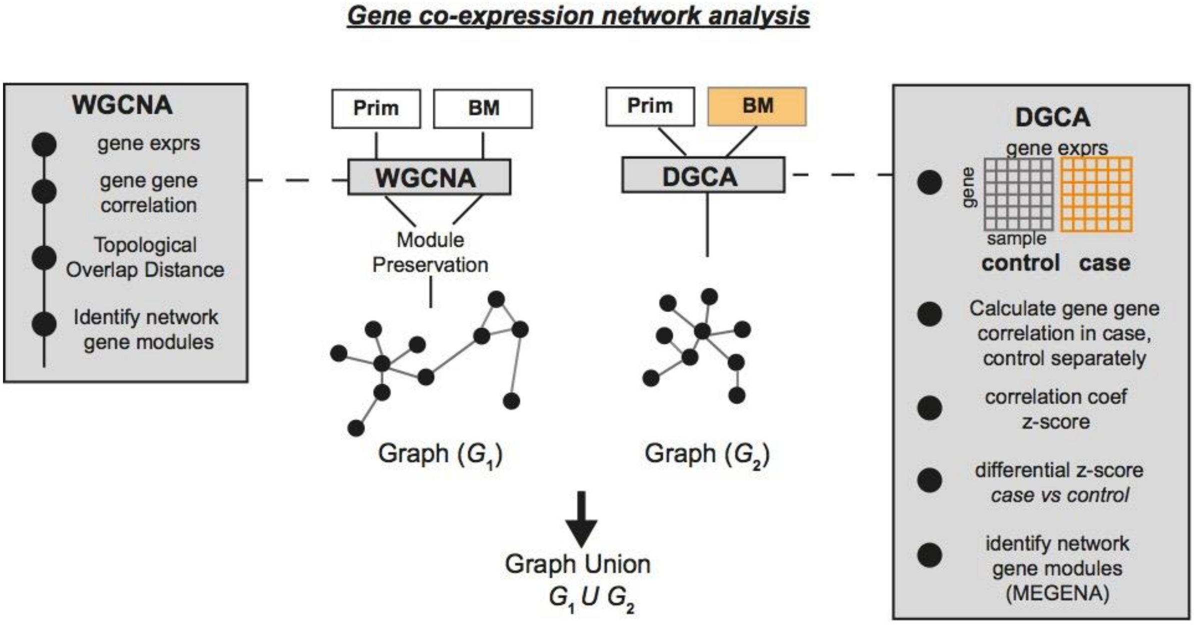

Supplementary Figure 4: Method summary for gene co-expression network analysis in brain metastases.

Weighted gene co-expression network analysis (WGCNA)(left) was used to identify gene co-expression networks separately in primary breast tumours (Prim) and brain metastatic tumours (BM) followed by a module preservation analysis. Module preservation analysis identifies which Prim gene modules are preserved in BM gene modules, represented by Graph $G_{1.}$ Differential gene co-expression analysis (DGCA) (right) identifies differential gene co-expression and associated gene network modules using MEGENA, in a case-control design analysis. Using DGCA and MEGENA, gene modules enriched in BM (orange label, case) compared to Prim (control) were identified, represented by Graph $G_{2.}$ The union of Graph G_1 and G_2 (middle bottom) represents the gene network that contains both preserved and enriched gene co-expression network modules in breast cancer brain metastases.

Gene co-expression networks

Group	# genes Primary breast samples*	# genes brain mets samples*	# prim modules	# brain mets modules	# preserved modules	# genes in preserved modules
**All tumours (N=45)**			17	28	**8**	**716**
**Luminal (N=13 patients)**	5,092	7,207	43	54	**8**	**197**
**HER2 (N=16 patients)**	4,007	4,559	50	57	**9**	**231**
**TNBC (N=16 patients)**	2,676	4,393	27	33	**4**	**229**

*SD>0.75

Supplementary Figure 5: Summary of results from weighted gene co-expression network analysis (WGCNA).
WGCNA method was used to identify subtype specific gene co-expression networks, separately for primary breast tumours and brain metastatic tumours, from gene expression data (gene selection based on standard deviation (SD > 0.75) used as input for WGCNA). For each subtype, the number of gene co-expression network modules identified are listed for all groups. WGCNA Module preservation analysis identified 8 (n=197 genes), 9 (n=231 genes) and 4 (n=229 genes) preserved gene modules in Luminal, HER2+ and TNBC molecular subtype in BCBM.

Differential gene co-expression networks

a

ER+/HER2- (n=13 patients)

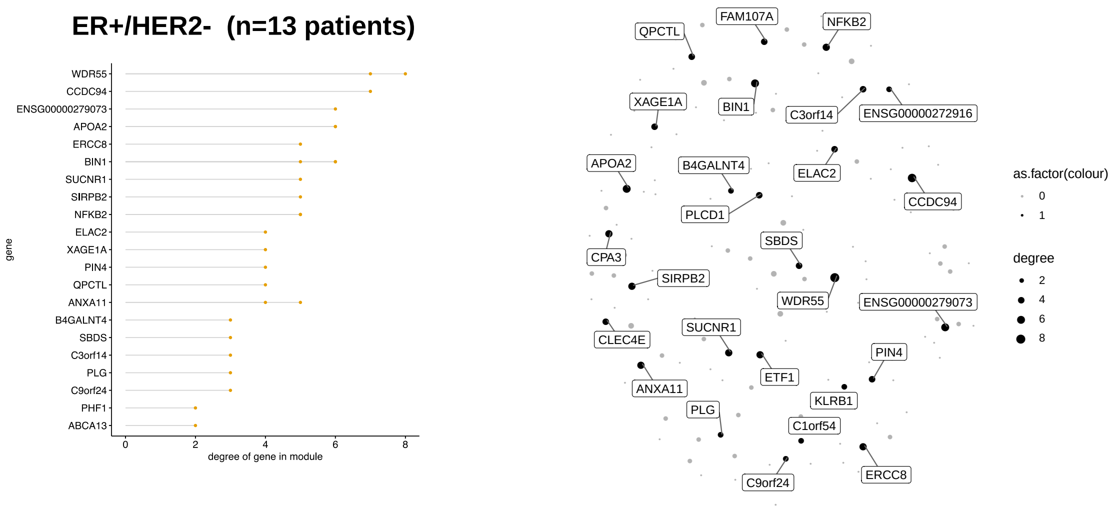

b

HER2+ (n=16 patients)

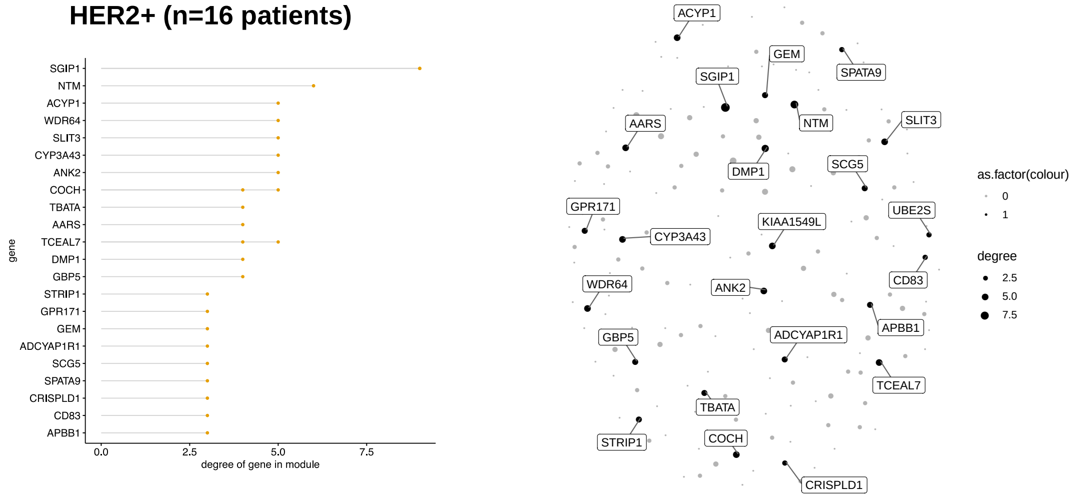

c

TNBC (n=16 patients)

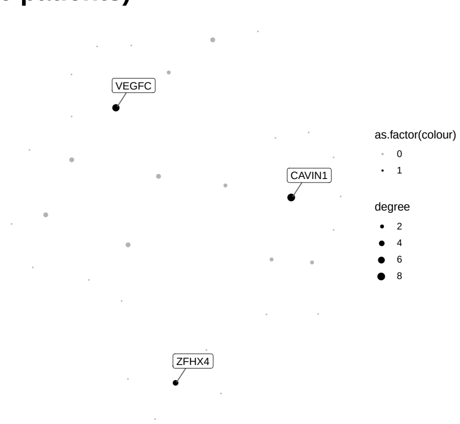

Supplementary Figure 6: Subtype specific differential gene co-expression networks in BCBM. (a-c) Dot plot of hub genes and their associated degree value for each differential gene co-expression network module identified for each subtype specific analysis: ER+/HER2-; HER2+; TNBC. Network plots labelled with hub genes with node size proportional to node degree value.

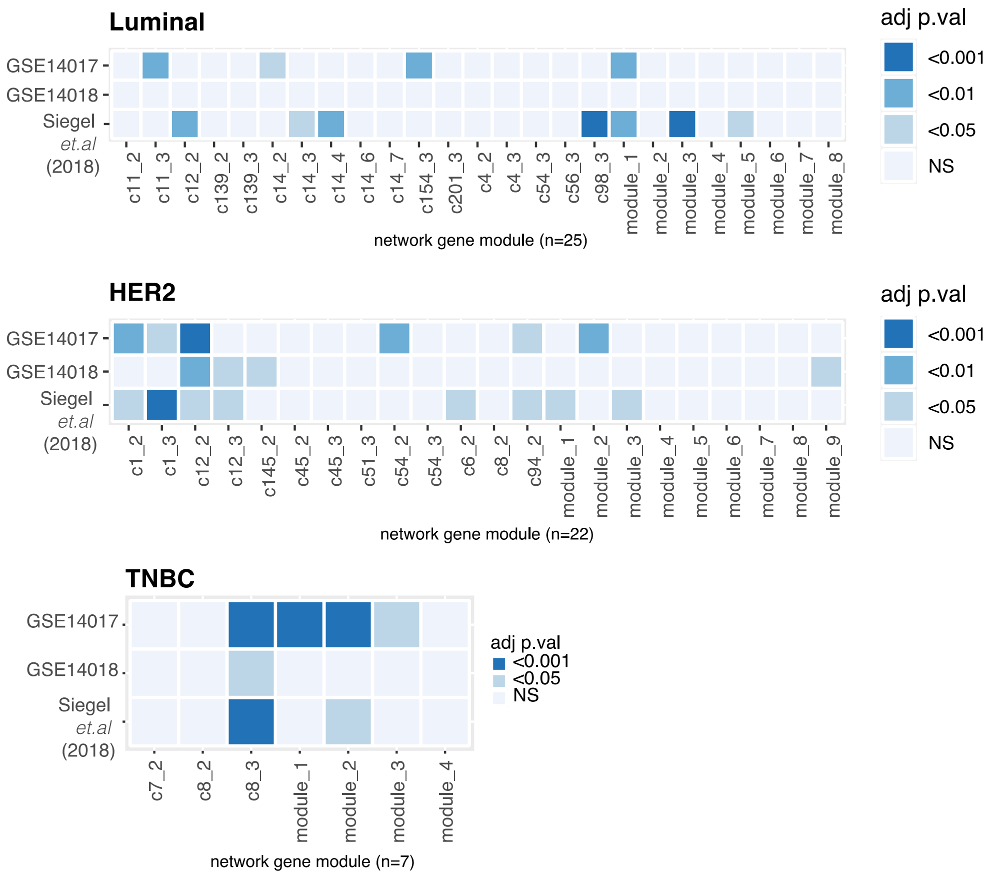

Supplementary Figure 7: Gene network module ranking by independent single sample enrichment testing in previously published breast cancer metastases gene expression data. Tileplot per subtype specific testing of gene network modules, coloured according to Benjamini Hochberg (BH) adjusted p-value threshold (<0.001, <0.01,<0.05 or NS: not significant) in three multiorgan breast cancer metastases datasets. Two-sided Wilcoxon rank-sum test was used to test if ssGSEA score for each gene module was significantly different (Benjamini Hochberg (BH) adjusted p-value < 0.05) in brain metastases versus all other metastatic tumour scores. The ggplot2 geom tile_plot() was used to visualise results.

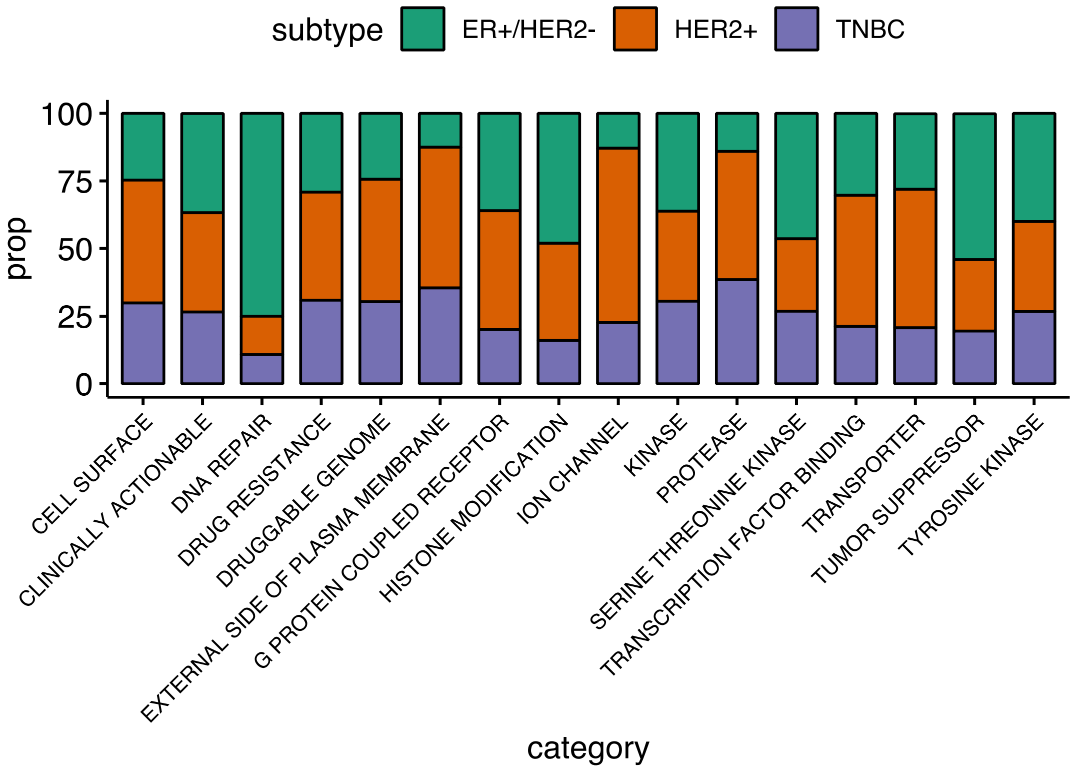

Supplementary Figure 8: Co-expression module genes categorized by both subtype and DgIdb gene categories.
Stacked bar chart of the relative proportion of the co-expression module genes for each clinical subtype characterised by the Drug-Gene Interaction database (DGidb) categories. Annotating co-expression module genes according to the DGidb categories revealed the highest proportion of DNA repair genes belonged to the luminal subtype network genes. prop=proportion. Source data are provided as a Source Data file.

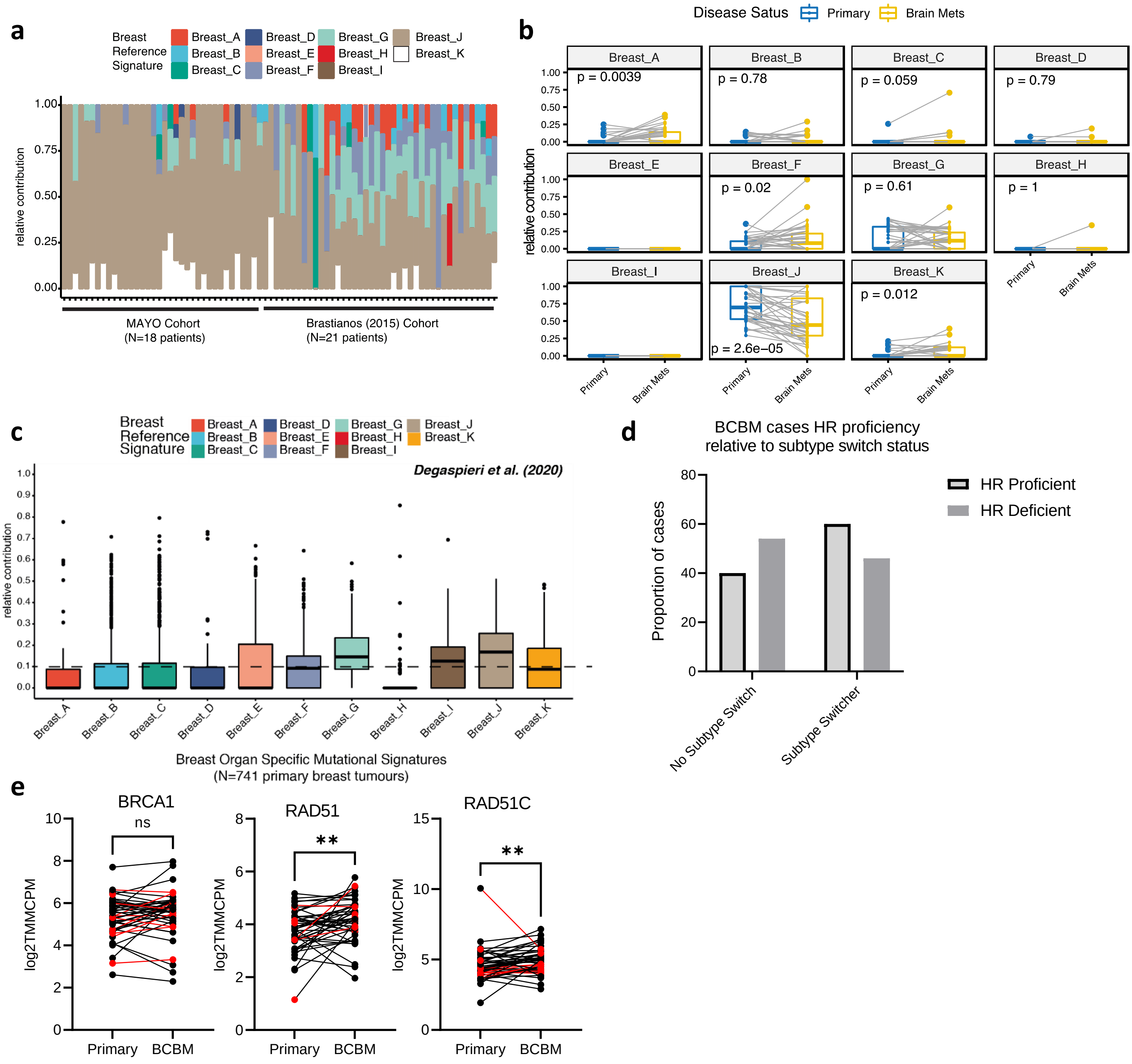

Supplementary Figure 9: Mutational signature profile all tumors.

(a) Stacked barchart of the relative contribution of mutational signatures detected in all primary breast and brain metastatic tumors for the MAYO Clinic (18 cases) and Brastianos et. al. (2015) (21 cases) WXS Cohorts. **(b)** Boxplot of the relative contribution of each mutational signature Breast A-K in patient-matched primary and brain metastatic tumors (39 cases). Paired two-sided Wilcoxon Rank Sum test performed on each tumor pair to test if relative contribution of each signature significantly increased or decreased in brain metastases compared to primary breast (p-value annotated on each boxplot). The upper and lower limits of the box correspond to the 1st and 3rd quartile of the relative contribution value distribution for each mutational signature respectively, with whiskers extending to 1.5 times the range from top/bottom of the box. **(c)** Boxplot of the relative contribution [0-1] of breast cancer specific reference mutational signatures (Breast A-K) detected in primary breast tumor samples (left-right) from Degasperi et al (N=741 patients). Median relative contribution value of Breast K 0.09 was used as a cutoff for HR proficiency (indicated by a dashed line in the graph). interquartile The upper and lower limits of the box correspond to the 1st and 3rd quartile of the relative contribution value distribution for each mutational signature respectively, with whiskers extending to 1.5 times the range from top/bottom of the box. **(d)** Bar chart representation of the proportion of BCBM cases (N=18) that are HR deficient or HR proficient according to the subtype switch status. **(e)** Paired ladder plot of BRCA1, RAD51 and RAD51C mRNA expression in patient-matched primary (P) and brain metastasis (BCBM) cases (n=45 patients; n=90 tumors) (log2norm CPM). Red dots represent tumors where HRD was detected based on the Breast K mutational signature presence. P value obtained via two-sided Wilcoxon signed-rank test. (BRCA1, P=0.19; RAD51, P=0.005; RAD51c, P=0.003). Source data are provided as a Source Data file.

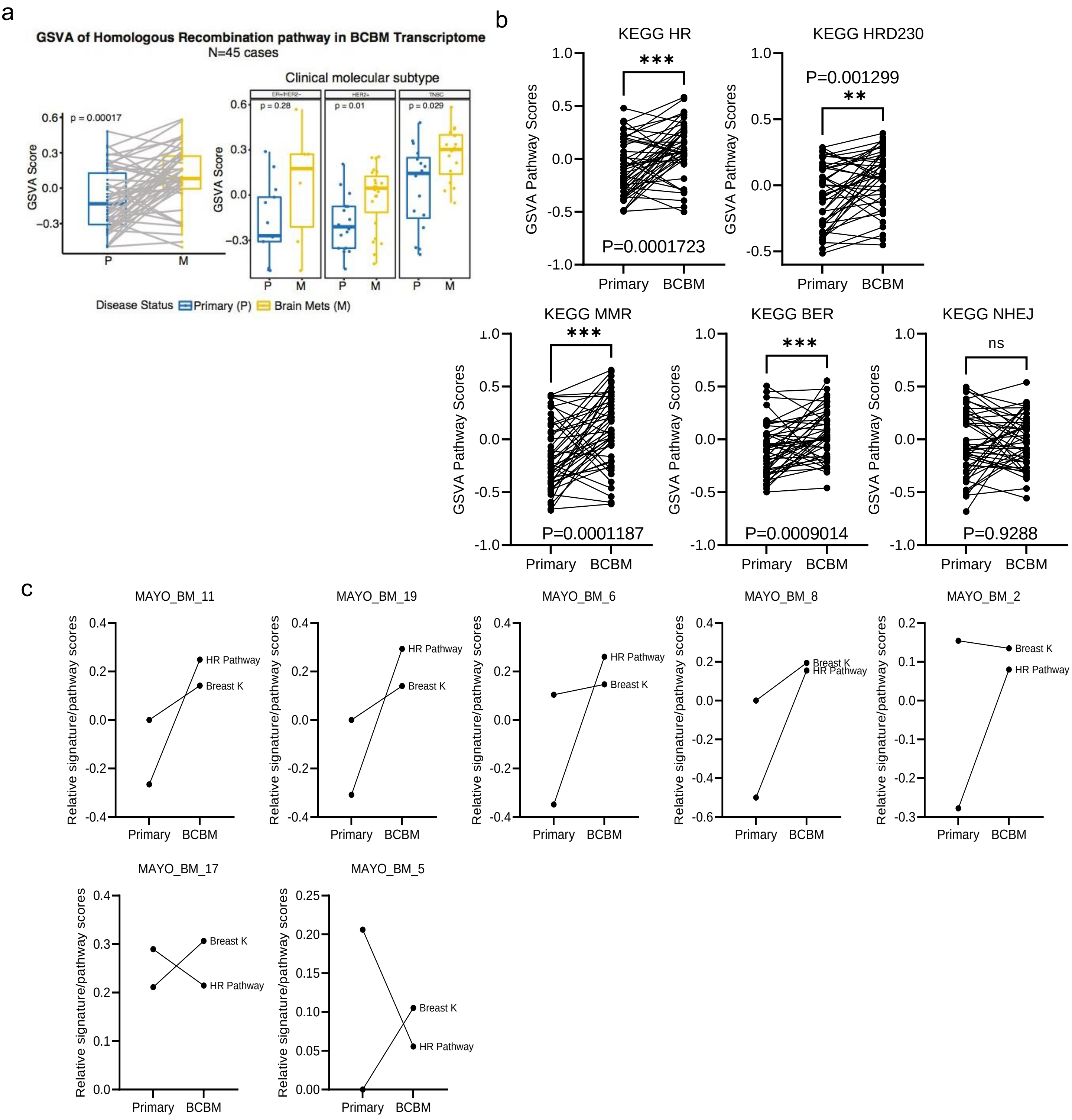

Supplementary Figure 10: HRD related signatures detected with RNA profiling.
(a) Boxplots of GSVA pathway score calculated from RNA-Seq of N=45 patients with BCBM, using genes in the homologous recombination (HR) pathway from KEGG database. Comparison of HR GSVA scores in patient matched tumor's (primary breast (blue) and brain metastatic tumor (yellow)) (paired two-sided Wilcoxon test All patients P=0.00017; ER+/HER2-, P=0.28; HER2+ve, P=0.01; TNBC, P=0.029). The upper and lower limits of the box correspond to the 1st and 3rd quartile of the GSVA pathway score distribution with whiskers extending to 1.5 times the range from top/bottom of the box. **(b)** Paired ladder plot of DNA-based HRD related signature Breast K alongside the RNA-seq based GSVA HR pathway for individual cases of primary and patient-matched brain metastasis (BCBM). (n=45 pairs; n=90 samples). P value obtained via two-sided Wilcoxon signed-rank test (HR,P=0.0001723; HRD230, P=0.0012999; MMR, P=0.0001187; BER, P=0.0009014; NHEJ, P=0.9288). **(c)** Paired ladder plot of HRD related signatures reported in old Figure 5b. GSVA pathways scores are plotted for the n=45 pairs of primary breast tumors (Primary) and brain metastasis (BCBM). Source data are provided as a Source Data file.

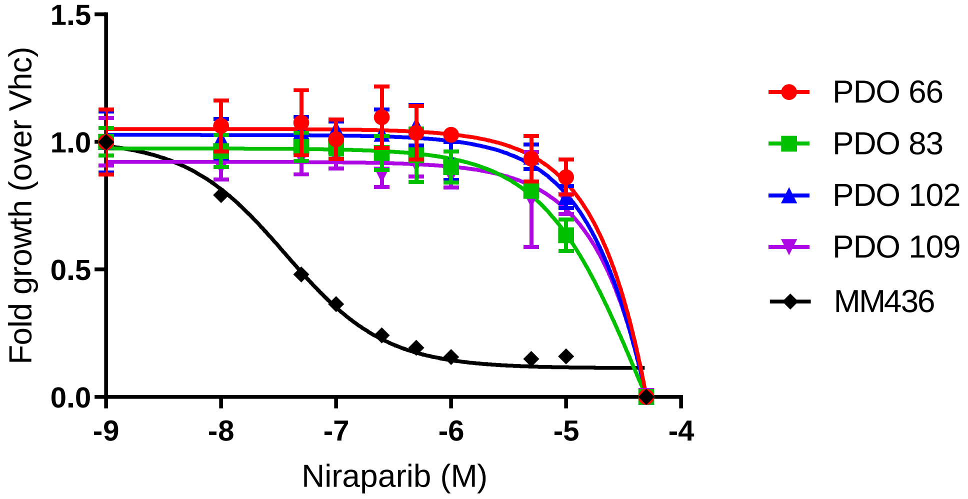

Supplementary Figure 11:. Niraparib response in patient-derived organoids.
A dose response curve for niraparib (0-5μM) carried out for Breast K high/Breast G negative T638org and BREAST K low/ Breast G high HCI05org models. (n=6-8 biologically independent organoids). Error bars represent mean ± SD. Source data are provided as a Source Data file.

Supplementary Methods

Gene Filtering and Normalisation

Genes with little to no expression across all 90 samples were filtered for subsequent analysis, according to the filtering strategy used previously[1]. In brief, if gene TPM > 0.5 in at least 10% of all samples (N=90), it was retained. The filtered TPM was utilised for any analysis dependent on a between sample comparison i.e. hierarchical clustering, gene set enrichment analysis (GSEA),gene set variation analysis (GSVA). Filtered gene level counts were normalised with edgeR *calcNormFactors()* and *cpm*() function, where counts per million (CPM): $CPM = C * 10^6/N$ *(counts* (C) scaled by the library depth in million units*)*. A log2 transformation was applied to CPM: *log2(CPM +1)*, where 1 is a pseudocount added to prevent negative value counts. Filtered log2 CPM and TPM genes were annotated using biomaRt R package to identify protein coding genes using the ENSEMBL GRCh38 p.10 database. Only protein coding genes were taken forward for downstream analysis.

Principal Component Analysis

Principal component analysis (PCA) reduces the dimensionality of multivariate data, to a number of orthogonal principal components (PC) which capture the largest overall sources of variation present in a dataset. PC's can be visualized graphically with minimal loss of information. PCA and *biplot* was used to assess which orthogonal sources of variation are due to batch effect or due to biological variation. Log2 DESeq2 normalised counts were used with *FactoMineR* (http://factominer.free.fr). To reduce the matrix size used for PCA, genes were first filtered to remove those which do not vary in expression (standard deviation (SD) < 0.25). Filtered and standardised (*scale = TRUE*), log2 normalised counts were used as input for PCA(), with output piped into biplot function for visualisation.

Batch effect correction

DESeq2 log2 variance stabilised transformation (VST) was applied to non-negative integer value, filtered protein coding gene expression counts, used as input to *svaseq* for all batch effect assessments[2]. A multiple linear regression model was fit for each surrogate variable (SV), with the following independent predictors: sequencing batch ID [1,5], disease status (primary breast (P) / brain metastases (M)), estrogen receptor (ER) IHC status (ER+/ER-) and primary tumour histological subtype (DCIS, IDC, ILC,

Mixed ILC/IDC). *Pre* and *post* batch correction evaluation was assessed using PCA, visualised as a biplot and unsupervised hierarchical clustering analysis.

A linear model fitted using SV1 was significantly associated with sequencing batch (labelled Centre)($P < 0.05$; R^2 ~0.70), with SV2,3 significantly associated with tumour subtype(Extended Data Fig.3). A batch driven effect was captured by PCA (Extended Data Fig. 2a). In order to correct for between RNA-Seq cohort (MAYO, PITT-RCSI) batch driven effect, we included SV1 as a covariate for batch in DESeq2 model formulae for differential gene expression testing. Otherwise, for other downstream analysis, where gene expression values were utilised (log2 DESeq2 normalised protein coding gene expression counts, log2 VST counts for network analysis and log2 TMM CPM normalised counts for plotting and TPM for GSEA, clustering and between sample comparisons), *removeBatchEffect*() function in LIMMA package in R, was used with SV1, to adjust gene expression values to account for mean shifts in expression driven by the batch driven effect.

Weighted gene co-expression network analysis (WGCNA)

The WGCNA method[3] was used to identify subtype specific gene co-expression networks separately for primary breast and brain metastatic tumors. Batch corrected log2 variance stabilized transformed (VST) gene expression counts, filtered by TPM, were used for correlation network analysis. Further stringent gene filtering was applied to remove genes with no variability of expression (standard deviation (SD) < 0.75) across all samples within a subtype specific analysis. A WGCNA wrapper function (https://github.com/joshua-d-campbell/utilities/blob/master/R/WGCNA_wrapper.R) was modified for use here, with default parameters except for the following adjustments: *corFnc = "bicor", minModuleSize=20, networkType="signed".* The *bicor* function was used to calculate a pairwise biweight mid correlation coefficient between gene pairs. The signed parameter was used to output a scaled correlation matrix. Typically, in a correlation coefficient matrix, values range from [-1,1] where -1 indicates a strong negative correlation between gene pairs; 0 there is no linear relationship and 1: a strong positive relationship. In the scaled correlation coefficient matrix, values range between [0,1] where < 0.5 indicate negative correlation and values > 0.5 indicate positive correlation. Next, the correlation matrix is transformed to an adjacency matrix, which represents a matrix of gene pairwise connection strengths, using a power function: *adjacency = (0.5 * (1 + cor))^soft_power*. A soft thresholding power was

chosen (R^2 > 0.9) based on an analysis of scale free topology for multiple soft thresholding powers for each network constructed. The soft thresholding approach emphasizes strong correlations, penalises weak correlations.

This signed network adjacency matrix was used to calculate the topological overlap distance matrix (TOM), which is a measure of gene interconnectedness based on how close neighbouring genes are to each other. A dissimilarity measure is calculated using (1-TOM) and used as a distance metric for clustering genes into groups, using the average linkage clustering algorithm. The *cuttreeDynamic* function is a variable height branch pruning technique used to select at which height to cut the dendrogram, to define gene co-expression network modules.The *minModuleSize* parameter was set to 20, meaning that each module has to contain at least 20 genes. Each module was assigned a unique colour (grey reserved for module 0, i.e., unassigned genes). Each module is represented by its first principal component known as a module eigengene (ME), which is a singular value which represents the highest % variance for all genes in a module. A Pearson correlation coefficient was calculated between each ME to identify strongly correlated MEs with highly similar expression patterns that could be merged, to arrive at the final set of modules in each gene co-expression network.

Gene module preservation analysis

Subtype specific gene co-expression modules maintained (preserved) in primary breast tumor and brain metastases were identified using two methods: 1) WGCNA modulePreservation method in R[4] and 2) as per Beane *et al* methodology[5]. Those modules identified as preserved by at least 1 method were used for subsequent analysis. Using the co-expressed gene modules identified in primary tumors as a reference set, and brain metastases identified modules as a test set, for each reference-test pair, *the modulePreservation()* function calculated module preservation statistics that measure how well the modules of the reference set are preserved in the test set. Parameters for *modulePreservation*: *bicor* correlation function; *networkType=signed*; number of permutations: 100; with a random seed set for reproducibility; max “gold” module size is 100 and max module size is 400. The *medianRank* and Z score summary module preservation statistics was plotted as a function of module gene size. Overall, the higher the value of Z score summary, the more preserved the module is between data sets: $5<Z<10$ indicates moderate

preservation, while Z>10 indicates high preservation. The "grey" module contains input genes which did not cluster into a distinct gene modules, while the gold module contains a set of random genes. Overall, the grey and gold modules should have lower Z-scores compared to moderately to highly preserved modules. A second method was applied for module preservation analysis Beane *et al* methodology[5]. In brief, gene sets were generated for each subtype specific gene co-expression module for the primary breast and brain metastatic tumor datasets and combined to create a compendium of gene sets. For each gene set in the compendium, the module eigengene (PC1) was calculated across each z-score normalized dataset (scale() function; center = T, scale = T). A matrix of absolute Pearson correlation coefficients based on PC1 values was calculated for each of the gene sets in the compendium. If r > 0.85, set to l; if r <= 0.85 set to 0. Matrices were summed and gene sets derived from brain metastatic co-expression modules correlated to other primary breast gene sets were retained. A correlogram of preserved gene co-expression gene modules were visualised using the corrplot R package for each molecular subtype analysed.

Differential gene co-expression network analysis

DGCA R package (v.1.0.2)[6] was used to detect differential co-expression (correlation) patterns between gene pairs in brain metastatic versus primary breast tumours in a subtype specific analysis. Similar to WGCNA, batch corrected log2 variance stabilised transformed (VST) protein coding gene expression counts, filtered by TPM, were used for correlation network analysis. Gene filtering was applied based on calculated variance: genes with gene expression variance > 25th percentile and expression values > 10th percentile was retained. For DGCA, gene pair Spearman correlation coefficients are calculated within each condition (here, the conditions are primary breast tumour and brain metastatic tumour), with statistically significant gene pairs retained (Benjamini Hochberg (BH) adjusted p-value < 0.05). Gene pairs with absolute *r* > 0.99 are likely spurious associations and are removed. The Fisher z-transformation is applied to Spearman correlation coefficients in each condition in order to stabilise sample variance prior to differential correlation testing. The difference in gene pair correlation z-scores (*dz*) between brain metastases and primary is calculated by the square root of the standard errors <equation>. Using *dz,* a two-sided p-value is calculated using the standard normal distribution, followed by BH adjustment method to generate an FDR for each gene pair. Statistically significant differential gene co-

expression pairs were called in each subtype specific analysis: Luminal FDR < 0.10; HER2 and TNBC subtype comparisons: FDR < 0.30.

Next, MEGENA R package (v.1.4.1)[7] was used for differential gene co-expression network construction and module detection. A planar filtered network (PFN) was built using significantly differential correlated gene pairs for subtype specific analysis. Network weights were assigned based on absolute zscore difference for each significant gene pair and normalised to [0,1] range (*PFN$weight <- (PFN$weight/max(PFN$weight)) * 0.99999999*). The PFN was converted to an *igraph* R object (*directed = FALSE*). In order to identify differential gene co-expression network modules, multiscale clustering method in MEGENA was applied to the PFN *igraph* object with the following parameters: *module p-value = 0.05; hub gene p-value = 0.05; minSize=5, maxSize = 800, nPerm=100,* with a seed set for reproducibility when using permutation-based testing.

Supplementary References

1. Vareslija, D.*, et al.* Transcriptome Characterization of Matched Primary Breast and Brain Metastatic Tumors to Detect Novel Actionable Targets. *J Natl Cancer Inst* **111**, 388-398 (2019).
2. Leek, J.T. svaseq: removing batch effects and other unwanted noise from sequencing data. *Nucleic Acids Res* **42**(2014).
3. Zhang, B. & Horvath, S. A general framework for weighted gene co-expression network analysis. *Stat Appl Genet Mol Biol* **4**, Article17 (2005).
4. Langfelder, P., Luo, R., Oldham, M.C. & Horvath, S. Is my network module preserved and reproducible? *PLoS Comput Biol* **7**, e1001057 (2011).
5. Beane, J.E.*, et al.* Molecular subtyping reveals immune alterations associated with progression of bronchial premalignant lesions. *Nat Commun* **10**, 1856 (2019).
6. McKenzie, A.T., Katsyv, I., Song, W.M., Wang, M. & Zhang, B. DGCA: A comprehensive R package for Differential Gene Correlation Analysis. *BMC Syst Biol* **10**, 106 (2016).
7. Song, W.M. & Zhang, B. Multiscale Embedded Gene Co-expression Network Analysis. *PLoS Comput Biol* **11**, e1004574 (2015).
