# Supplementary material for: Mapping molecular subtype specific alterations in breast cancer brain metastases identifies clinically relevant vulnerabilities
Source: Nat Commun. 2022 Jan 26;13:514. doi: 10.1038/s41467-022-27987-5 (PMC8791982; doi:10.1038/s41467-022-27987-5)
Supplement: Supplementary file 2 — Description of the Additional Supplementary Files [file 41467_2022_27987_MOESM2_ESM.pdf]

Description of the Additional Supplementary Files

Title: Supplementary Data 1
Description: Clinico-pathological characteristics of Exome Capture RNA-Seq Cohort (45 patients; N=90 samples). (.xlsx)

Title: Supplementary Data 2
Description: PAM50 Intrinsic molecular subtype of 90 samples in RNA-Seq Cohort. (.xlsx)

Title: Supplementary Data 3
Description: Results of differential gene expression testing (brain mets vs primary breast tumour) for Luminal patients (ER+/HER2-). Significant DEG (+/- log2 Fold Change 1.5 ; adjusted p-value < 0.01), table sorted by adjusted pvalue and "Set". (.xlsx)

Title: Supplementary Data 4
Description: Results of differential gene expression testing (brain mets vs primary breast tumour) for HER2+ subtype. Significant DEG (+/- log2 Fold Change 1.5 ; adjusted p-value < 0.01), table sorted by adjusted pvalue and "Set". (.xlsx)

Title: Supplementary Data 5
Description: Results of differential gene expression testing (brain mets vs primary breast tumour) for TNBC subtype. Significant DEG (+/- log2 Fold Change 1.5 ; adjusted p-value < 0.01), table sorted by adjusted pvalue and "Set". (.xlsx)

Title: Supplementary Data 6
Description: List of significantly differentially expressed genes (log2FC+/-1.5; p-value < 0.01) in BM vs primary tumour, common to all molecular subtypes. (.xlsx)

Title: Supplementary Data 7
Description: GSEA output (FDR < 0.25; NES +/-1.0) for MSigDB pathways analysed for significantly differentially expressed genes (log2FC+/-1.5; p-value < 0.01) in BM vs primary tumour, common to all molecular subtypes. (.xlsx)

Title: Supplementary Data 8
Description: GSEA output (FDR < 0.25; NES +/-1.0) for MSigDB pathways analysed for subtype specific (Luminal, HER2+, TNBC) significantly differentially expressed genes (log2FC+/-1.5; p-value < 0.01) in BM vs primary tumour. (.xlsx)

Title: Supplementary Data 9
Description: single sample GSEA (ssGSEA) scores for all subtype specific network modules tested in independent datasets of breast cancer metastatic tumours. (.xlsx)

Title: Supplementary Data 10
Description: GSEA output (FDR < 0.25; NES +/-1.0) for MSigDB pathways analysed for RNA-Seq subtype specific (Luminal, HER2+, TNBC) gene co-expression network modules found to be brain specific. (.xlsx)

Title: Supplementary Data 11
Description: Details of the WXS BCBM Cohort (N=39 patients). (.xlsx)

Title: Supplementary Data 12
Description: GISTIC Arm (Broad) level events for patient matched primary breast and brain metastatic tumours. Columns referencing Amplification related values in red and Deletions in blue with significant alterations highlighted in dark grey (q value). (.xlsx)

Title: Supplementary Data 13
Description: Focal somatic copy number gains and high level amplifications indentified by GISTIC2.0 (v2.0.23) as recurrent across primary breast and brain metastatic tumours, from WXS Breast Cancer Brain Metastases Cohort (N=39 patients). (.xlsx)

Title: Supplementary Data 14
Description: Focal somatic copy number loss and homozygous deletions indentified by GISTIC2.0 (v2.0.23) as recurrent across primary breast and brain metastatic tumours, from WXS Breast Cancer Brain Metastases Cohort (N=39 patients) . (.xlsx)

Title: Supplementary Data 15
Description: SNV's , INDELs and somatic copy number alterations (SCNA) in co mutation plot for brain metastatic tumours from WXS BCBM Cohort (N=39 patients). dNdSCV significant SNV's in brain metastatses; Tumour mutational burden (TMB) shown. (.xlsx)

Title: Supplementary Data 16
Description: Matrix of relative contribution values for mutational signtaures Breast A-K in WXS BCBM Cohort (N=39 primary breast; N=39 brain metastatic tumour). (.xlsx)

Title: Supplementary Data 17
Description: Samples listed highlighting those where homologous recombination deficiency (HRD) associated mutational signature Breast K was detected (HRD_mut_sig= Deficient/ Green colour). Also provided are specific annotations for the genomic scar scores. (.xlsx)

Title: Supplementary Data 18
Description: gBRCA1/2 and PALB2 mutations called from normal samples of WXS BCBM Cohort (N=39 patients). Those variants annotated as IMPACT= HIGH/MODERATE and ClinVar database. (.xlsx)

Title: Supplementary Data 19
Description: GSVA Pathway Scores for DNA repair pathways calculated from RNA-Seq (N=90 samples). tumourID: Primary breast tumour (P), brain metastatic tumour (M). (.xlsx)

Title: Supplementary Data 20
Description: Detailed description of the patient-derived xenograft and organoid cohort. (.xlsx)
