# Supplementary material for: Mapping molecular subtype specific alterations in breast cancer brain metastases identifies clinically relevant vulnerabilities
Source: Nat Commun. 2022 Jan 26;13:514. doi: 10.1038/s41467-022-27987-5 (PMC8791982; doi:10.1038/s41467-022-27987-5)
Supplement: Supplementary file 23 — Reporting Summary [file 41467_2022_27987_MOESM23_ESM.pdf]

Corresponding author(s): Leonie S Young, Fergus Couch

Last updated by author(s): Dec 15, 2021

Reporting Summary

Nature Portfolio wishes to improve the reproducibility of the work that we publish. This form provides structure for consistency and transparency in reporting. For further information on Nature Portfolio policies, see our Editorial Policies and the Editorial Policy Checklist.

Statistics

For all statistical analyses, confirm that the following items are present in the figure legend, table legend, main text, or Methods section.

n/a	Confirmed	
☐	☒	The exact sample size (*n*) for each experimental group/condition, given as a discrete number and unit of measurement
☐	☒	A statement on whether measurements were taken from distinct samples or whether the same sample was measured repeatedly
☐	☒	The statistical test(s) used AND whether they are one- or two-sided<br>*Only common tests should be described solely by name; describe more complex techniques in the Methods section.*
☐	☒	A description of all covariates tested
☐	☒	A description of any assumptions or corrections, such as tests of normality and adjustment for multiple comparisons
☐	☒	A full description of the statistical parameters including central tendency (e.g. means) or other basic estimates (e.g. regression coefficient) AND variation (e.g. standard deviation) or associated estimates of uncertainty (e.g. confidence intervals)
☐	☒	For null hypothesis testing, the test statistic (e.g. *F*, *t*, *r*) with confidence intervals, effect sizes, degrees of freedom and *P* value noted<br>*Give P values as exact values whenever suitable.*
☒	☐	For Bayesian analysis, information on the choice of priors and Markov chain Monte Carlo settings
☒	☐	For hierarchical and complex designs, identification of the appropriate level for tests and full reporting of outcomes
☐	☒	Estimates of effect sizes (e.g. Cohen's *d*, Pearson's *r*), indicating how they were calculated

Our web collection on statistics for biologists contains articles on many of the points above.

Software and code

Policy information about availability of computer code

Data collection

DNA Seq
- hg19 / GRCh37 human reference genome
- bwa mem v.0.7.13
- GATK4 v.4.1.2.0
- Picard v.1.140
- Mutect2 v.4.1.2
- Strelka v. 2.9.8
- bcftools v.1.9-40
- ngs filter v1.4 (https://github.com/mskcc/ngs-filters)
- snp-pileup v.0.6.1
- FACETS v.0.6.1
- FACETS Suite v 2.0.8
- GISTIC2.0 v.2.0.23
- Signal v.1.0 (https:// signal.mutationalsignatures.com/analyse)
- GATK HaplotypeCaller v. 4.1.2
- vcf2maf v1.6.17
- coMut v.0.0.3 (https://github.com/vanallenlab/comut)

RNA Seq
- GRCh38.p10 (GENCODE v.27) human reference transcripts
- BBDuk v.38.0
- Salmon v.0.91
- STAR v.2.6.1a
- samtools v.1.9

- RSeQCv.2.4.0
- MultiQC v.1.7
- SankeyMATIC v.0.0.0 (https://sankeymatic.com/)
- Cytoscape v.3.7- EnrichmentMap plugin

Data analysis

No custom codes were used in the analysis reported in this study. All relevant references are provided in the methods section.
All data analysis using R v.3.6.0 to v.3.6.3
R package versions:
- dndscv v.0.0.1.0
- genefu v.2.18.1
- ComplexHeatmap v.2.2.0
- DGCA v.1.0.2
- MEGENA v.1.4.1
- maftools v.2.4.10
-tximport v.1.22.0
-DESeq2 v.1.26.0
-WGCNA v.1.69
-WGCNA wrapper function (https://github.com/joshua-d-862 campbell/utilities/blob/master/R/WGCNA_wrapper.R)
-fgsea v.1.12.0
- GSVA v.1.34.0
-edgeR v.3.28.1
-biomaRt v.2.42.1
-FactoMineR v.2.3 (http://factominer.free.fr)
-sva (svaseq) v.3.34.0
-limma v.3.42.2
-GEOquery v.2.54.1
-cluster v.2.1.0
- corrplot v.0.84
-viridis v. 0.5.1
-ggpubr v.0.3.0
-ggplot2 v.3.3.3

Other Software:
Adobe Illustrator 2019 v.23.1.1

For manuscripts utilizing custom algorithms or software that are central to the research but not yet described in published literature, software must be made available to editors and reviewers. We strongly encourage code deposition in a community repository (e.g. GitHub). See the Nature Portfolio guidelines for submitting code & software for further information.

Data

Policy information about availability of data

All manuscripts must include a data availability statement. This statement should provide the following information, where applicable:

- Accession codes, unique identifiers, or web links for publicly available datasets
- A description of any restrictions on data availability
- For clinical datasets or third party data, please ensure that the statement adheres to our policy

In line with Institutional Review Board approvals from all three participating Institutions including University of Pittsburgh, Royal College of Surgeons in Ireland and Mayo Clinic, raw RNA (N=45patients/N=90 breast cancer brain metastasis cases) and WES DNA (N=18 matched normal, primary breast and brain metastatic tumour) data was not deposited in a public repository as informed consent was not available with these samples. Raw RNA and DNA sequencing data for the paired primary and metastatic samples will be made available upon request and under regulatory compliance via a data usage agreement (DUA). Please contact the corresponding author with data access requests that will be granted once the DUA is signed. Processed RNA sequencing data for all cases reported in the study (N=45patients/ N=90 breast cancer brain metastasis cases) is deposited in the Gene Expression Omnibus under the accession number GSE184869 [https://www.ncbi.nlm.nih.gov/geo/query/acc.cgi?acc=GSE184869]. For the WES DNA (N=18 matched normal, primary breast and brain metastatic tumour) samples newly generated as part of the study, the processed files are available on figshare [https://doi.org/10.6084/m9.figshare.16685680.v1]. WES data for 21 of the 39 breast cancer brain metastases cases (matched normal, primary breast and brain metastatic tumour) has been described previously and are available to downloaded upon request from the database of Genotypes and Phenotypes (dbGap) (accession number phs000730.v1.pl) [https://www.ncbi.nlm.nih.gov/projects/gap/cgi-bin/study.cgi?study_id=phs000730.v1.p1]. RNA-Seq data from Siegel et al.14 (N=16 patients; 68 metastases) were downloaded from the dbGaP (accession number phs000676) [https://www.ncbi.nlm.nih.gov/projects/gap/cgi-bin/study.cgi?study_id=phs000676.v1.p1]. Supplementary table 4 from the Rinaldi et al., (2020) targeted sequencing study of approx. 11,000 unmatched primary breast, local recurrence and distant metastatic tumours using the FoundationOne assay is available at [https://doi.org/10.1371/journal.pone.0231999]. For GSEA the molecular signature database (MSigDB v.6.2) is available at [https://www.gsea-msigdb.org/gsea/msigdb]. The 230 member gene signature associated with homologous recombination deficiency (HRD230) was obtained from [https://www.nature.com/articles/ncomms4361#Sec22]. Network genes were cross referenced against genes in the "DNA Repair" category of the Drug-Gene Interaction database [https://www.dgidb.org/] version 3.0 (DGIdb 3.0). The microarray derived gene expression data for the multi organ breast metastatic tumours is available for download on GEO using the accession IDs: GSE14017 [https://www.ncbi.nlm.nih.gov/geo/query/acc.cgi?acc=GSE14017] and GSE14018 [https://www.ncbi.nlm.nih.gov/geo/query/acc.cgi?acc=GSE14018]. Source data are provided with this paper.

Field-specific reporting

Please select the one below that is the best fit for your research. If you are not sure, read the appropriate sections before making your selection.

☒ Life sciences ☐ Behavioural & social sciences ☐ Ecological, evolutionary & environmental sciences

For a reference copy of the document with all sections, see nature.com/documents/nr-reporting-summary-flat.pdf

Life sciences study design

All studies must disclose on these points even when the disclosure is negative.

Sample size	No statistical method was used to predetermine sample size. Sample size was determined by availability of matched primary breast and brain metastatic tumor tissue for sequencing. Sample size was considered to be sufficient given the difficulty in obtaining matched tumor tissue from patients with breast cancer brain metastases across all clinically relevant tumor subtypes.
Data exclusions	No samples analyzed were excluded.
Replication	For patient sequencing studies replication of each individual sample is not possible and is not utilized in standard practice. In in vitro studies with organoids 6-8 biological replicates per experiment were employed. All attempts at replication were successful for in vitro/ex vivo studies.
Randomization	Randomization was not performed as sequenced samples are from a retrospective non interventional observational study. The primary objective of this study was to better characterize the genomic and transcriptomic alterations that are observed in brain metastases from breast cancer across all clinically relevant tumor subtypes. For subtype specific analysis, samples were assigned to groups based on their known clinical status. For this observational cohort, controlling for covariates from non-random allocation of samples was not explicitly performed, other than for differential gene expression testing using DESeq2 where covariates were included to a) control for between sequencing center batch effect and b) to control for patient specific variation as these were patient matched samples. This is described in the methods and extended data for the RNA sequencing data.
Blinding	Blinding was only performed for immunohistochemical analysis where the investigators analysing and scoring images were blinded to account for potential observer bias when counting number of positive stained cells. Otherwise blinding was considered not necessary given samples were not collected as part of an interventional randomized controlled clinical trial.

Reporting for specific materials, systems and methods

We require information from authors about some types of materials, experimental systems and methods used in many studies. Here, indicate whether each material, system or method listed is relevant to your study. If you are not sure if a list item applies to your research, read the appropriate section before selecting a response.

Materials & experimental systems

n/a	Involved in the study
☐	☒ Antibodies
☐	☒ Eukaryotic cell lines
☒	☐ Palaeontology and archaeology
☐	☒ Animals and other organisms
☐	☒ Human research participants
☒	☐ Clinical data
☒	☐ Dual use research of concern

Methods

n/a	Involved in the study
☒	☐ ChIP-seq
☒	☐ Flow cytometry
☒	☐ MRI-based neuroimaging

Antibodies

Antibodies used	RAD51 (1:200; mouse monoclonal, Genetex, GTX70230; Clone 14B4, Isotype IgG2b) and ki67 (1:50; mouse monoclonal; Dako, M7240; MIB-1 clone, IgG1, kappa).
Validation	• RAD51 (1:200; mouse monoclonal, Genetex, GTX70230; Clone 14B4, Isotype IgG2b). Immunohistochemistry (IHC) specific validation carried out in-house. The staining was carried out with an automated Dako system and with an optimised antibody dilution. The manufacturer's website indicates that the antibody is IHC validated but the suggested dilution is assay dependent. Manufacturer IHC-specific reference: Chopra N et al. Nat Commun 2020; 11 (1):2662 Homologous recombination DNA repair deficiency and PARP inhibition activity in primary triple negative breast cancer.<br>• ki67 (1:50; mouse monoclonal; Dako, M7240; MIB-1 clone, IgG1, kappa). With more than 4000 literature citations, the MIB-1 antibody has now been established as an important monoclonal mouse antibody for the demonstration of the Ki-67 antigen in formalin-fixed, paraffin-embedded specimens. In Western blotting of lysates of the multiple myeloma cell line, IM-9, the MIB-1 antibody labels bands of 345 and 395 kDa, identical to the bands labeled by the original Ki-67 antibody. Scholzen T, Gerdes J. The Ki-67 protein: from the known and the unknown [review]. J Cell Physiol 2000,182:311-22.

Eukaryotic cell lines

Policy information about cell lines

Cell line source(s)	MDA-MB-436 (ATCC)
Authentication	Cells used are authenticated using STR DNA typing and is consistent with the International Cell Line Authentication Committee guidelines (SourceBioScience).
Mycoplasma contamination	Cells are regularly tested for mycoplasma contamination (LT07-118, Lonza) and these cell lines were mycoplasma negative.
Commonly misidentified lines (See ICLAC register)	n/a

Animals and other organisms

Policy information about studies involving animals; ARRIVE guidelines recommended for reporting animal research

Laboratory animals	female NOD-SCID (NOD.CB17-Prkdc<scid>/NcrCrl)
Wild animals	N/A
Field-collected samples	N/A
Ethics oversight	All procedures using animals were reviewed and approved by the Institutional Animal Care and Use Committee and the HPRA.

Note that full information on the approval of the study protocol must also be provided in the manuscript.

Human research participants

Policy information about studies involving human research participants

Population characteristics	The study population involves female breast cancer patients from three independent institutions, not pre-selected. A description of the covariate relevant study population and tumor characteristics including age, clinical tumor subtypes, pre- and post-menopausal status age groups, lines of treatment and other clinical characteristics can be found in Supplementary Data 1.
Recruitment	Only criteria were that patients had primary breast cancer and had subsequently developed brain metastasis. Only patients with FFPE tissue available for both primary breast and brain metastatic tumors were eligible to be included in the sequencing study. The only criteria for studies where freshly resected tissue samples were collected for ex vivo studies was that the brain metastasis were resectable as part of routine clinical care and that they were of breast cancer origin. Our study (both sequencing and ex vivo) are biased towards brain metastasis that can be resected or biopsied when clinically indicated.
Ethics oversight	Institutional review boards from all three participating Institutions University of Pittsburgh, Royal College of Surgeons in Ireland and Mayo Clinic approved collection and analysis of specimens. For sequencing studies, requirement for informed consent was waived, considering all samples were de-identified, there was no more than minimal risk to human subjects, and all tissue was obtained as part of routine clinical care. Freshly resected breast cancer brain metastatic tumors utilised in tumour explant and organoid studies were collected with fully informed consent from patients and studied under approved IRB protocol #13/09/ICORG09/07 at the Royal College of Surgeons in Ireland. All procedures using animals were reviewed and approved by the Institutional Animal Care and Use Committee and the HPRA.

Note that full information on the approval of the study protocol must also be provided in the manuscript.
